# Unveiling the Correlation between Inadequate Energy/Macronutrient Intake and Clinical Alterations in Volunteers at Risk of Metabolic Syndrome by a Predictive Model

**DOI:** 10.3390/nu13041377

**Published:** 2021-04-20

**Authors:** Francesca Danesi, Carlo Mengucci, Simona Vita, Achim Bub, Stephanie Seifert, Corinne Malpuech-Brugère, Ruddy Richard, Caroline Orfila, Samantha Sutulic, Luigi Ricciardiello, Elisa Marcato, Francesco Capozzi, Alessandra Bordoni

**Affiliations:** 1 Department of Agricultural and Food Sciences (DISTAL), University of Bologna, 47521 Cesena, Italy; francesca.danesi@unibo.it (F.D.); carlo.mengucci2@unibo.it (C.M.); simona.vita2@unibo.it (S.V.); francesco.capozzi@unibo.it (F.C.); 2 Interdepartmental Centre for Agri-food Industrial Research (CIRI Agrifood), University of Bologna, 47521 Cesena, Italy; 3 Department of Physiology and Biochemistry of Nutrition, Max Rubner-Institut, 76131 Karlsruhe, Germany; achim.bub@mri.bund.de (A.B.); stephanie.seifert@mri.bund.de (S.S.); 4 Unité de Nutrition Humaine (UNH), Université Clermont Auvergne, INRAE, CRNH Auvergne, F-63000 Clermont-Ferrand, France; corinne.malpuech-brugere@uca.fr; 5 Centre Hospitalier Universitaire (CHU) de Clermont Ferrand, CRNH Auvergne, F-63000 Clermont-Ferrand, France; ruddy.richard@udamail.fr; 6 School of Food Science and Nutrition, University of Leeds, Leeds LS2 9JT, UK; c.orfila@leeds.ac.uk (C.O.); s.sutulic@leeds.ac.uk (S.S.); 7 Gastroenterological Unit, Department of Medical and Surgical Sciences (DIMEC), University of Bologna, 40138 Bologna, Italy; luigi.ricciardiello@unibo.it (L.R.); e.marcato@tin.it (E.M.)

**Keywords:** metabolic syndrome, energy intake, macronutrient intake, penalised models, feature shrinkage, prevention

## Abstract

Although lifestyle-based interventions are the most effective to prevent metabolic syndrome (MetS), there is no definitive agreement on which nutritional approach is the best. The aim of the present retrospective analysis was to identify a multivariate model linking energy and macronutrient intake to the clinical features of MetS. Volunteers at risk of MetS (F = 77, M = 80) were recruited in four European centres and finally eligible for analysis. For each subject, the daily energy and nutrient intake was estimated using the EPIC questionnaire and a 24-h dietary recall, and it was compared with the dietary reference values. Then we built a predictive model for a set of clinical outcomes computing shifts from recommended intake thresholds. The use of the ridge regression, which optimises prediction performances while retaining information about the role of all the nutritional variables, allowed us to assess if a clinical outcome was manly dependent on a single nutritional variable, or if its prediction was characterised by more complex interactions between the variables. The model appeared suitable for shedding light on the complexity of nutritional variables, which effects could be not evident with univariate analysis and must be considered in the framework of the reciprocal influence of the other variables.

## 1. Introduction

Metabolic syndrome (MetS) is a pathologic condition including a cluster of components such as hypertension, dyslipidaemia, insulin resistance, hyperinsulinemia, glucose intolerance, and obesity, in particular central obesity [1]. MetS represents an epidemic clinical condition in countries where obesity and Western, unhealthy dietary patterns prevail, and its development is associated with both non-modifiable and modifiable risk factors as low physical activity and a poor-quality diet [2].

Currently, lifestyle-based interventions aimed at normalising body weight (BW) and controlling lipid levels, glucose sensitivity, and blood pressure are the most effective preventive approaches to MetS. Although available evidence suggests certain nutrients, foods, and dietary patterns have beneficial effects on MetS, there is no definitive agreement on which nutritional strategy is the most effective [3,4]. The association between different eating patterns and the MetS components has been evaluated in several studies [5,6]; in general, adherence to the Mediterranean or Nordic diets is associated with a lower prevalence of MetS or reduction in its components [7], while a Western dietary pattern is positively correlated with greater odds of MetS [8]. Conversely, the association between the individual macronutrient intake and the components of the MetS has been analysed in a few studies [9,10] and controversy still exists about the optimal amount and source of dietary macronutrients and their relative proportions to counteract MetS risk.

Over the past decades, an impressive body of quantitative knowledge regarding how dietary changes impact various aspects of BW and metabolism has been accumulated. Integrating this knowledge to make quantitative predictions is a formidable task given the multiple nonlinear interactions between various organ systems. Such an integrative approach is required to better connect energy and nutrient intake to normal physiology as well as to derangements that underlie conditions such as obesity, diabetes, and MetS.

To our knowledge, there are no available reports demonstrating the predictive role of the energy/macronutrient intake gaps, as assessed by the difference with the dietary reference values, on the clinical parameters related to MetS. In the present retrospective study, we correlated energy and macronutrient intake to the clinical features of MetS, with the final aim to provide an additional indication about the most important dietary contributors to clinical abnormalities related to an increased risk of MetS.

To grasp the role of each nutritional variable in the general frame of MetS pathological conditions, a model selection for various regression models between nutritional variables and clinical outcomes was performed. The analysis was inherently multivariate and allowed for the unveiling of how inadequate energy/macronutrient intake can predict clinical alterations leading to the MetS onset in a group of subjects at risk of the disease.

## 2. Materials and Methods

### 2.1. Participants

The subjects involved in the study were men and women (age 18–80 years) at risk for MetS enrolled in the randomised, double-blind, placebo-controlled, parallel intervention trial performed in the framework of the EU project PATHWAY-27. Eligible volunteers had two to four of the MetS diagnostic criteria [11], with at least one of them being elevated fasting triglycerides (TG) or low high-density lipoprotein cholesterol (HDL-C). Exclusion criteria are reported in Table S1. Volunteers were recruited in four European centres: Human Nutrition Research Centre of Auvergne (Clermont-Ferrand, France), Max Rubner-Institut (Karlsruhe, Germany), University of Leeds (Leeds, UK), and St. Orsola-Malpighi Hospital (Bologna, Italy).

The study was performed in full accordance with the ethical principles stated in the Declaration of Helsinki 1964, as revised in 2013 (Fortaleza, Brazil) [12]. Approval was obtained from the relevant local research ethics committees and additional regulatory bodies of the participating countries. All participants gave their written informed consent freely. Personal data were treated as strictly confidential by all persons involved in the trial. All data were collected and managed in a pseudonymised form, as previously reported [13]. Access to data was restricted to project partners, who receive only coded data for analysis.

At recruitment, blood pressure and anthropometric measurements (height, weight, and waist circumference, WC) were taken by trained staff as described in Bub et al. (2019) [13]. Blood was collected and analysed, as previously reported [14]. The present study addressed the intake of energy and macronutrients at baseline as possible dietary predictors of the onset of MetS. Clinical results of the intervention trial, as well as the possible predictive role of micronutrient intake, will be reported elsewhere.

### 2.2. Dietary Assessment

At recruitment, participants were asked to complete a validated semiquantitative food frequency questionnaire (FFQ) that was developed in the European Prospective Investigation into Cancer and Nutrition (EPIC) study [15], and a 24-h dietary recall (24hR), which is designed to assess energy and nutrient intake. The FFQ (covering a 12-month period) and the 24hR were administered by trained personnel.

Both FFQ and 24hR were completed by 281 participants (125 females and 156 males). Of the 281 dietary assessments, 66 with missing clinical information were excluded from the analysis and 215 subjects (94 females, aged 23–77 years, and 121 males, aged 24–78 years) having a complete dataset of both dietary assessment and clinical parameters were considered. After misreporting evaluation (see Section 2.2.1), 157 subjects were included in the analysis.

#### 2.2.1. Energy and Nutrient Intake and Misreporting

Energy and nutrient intakes from all foods and beverages were calculated using national and international databases and literature information. Dietary information by 24hR was used to corroborate energy and food intakes provided by the FFQ.

Daily energy intake was derived for each subject. Daily intake of total available carbohydrates, sugars, total fat, saturated fat, and unsaturated fat was expressed as percentage of daily energy intake (%EI). Intake of protein, dietary fibre, and alcohol was reported as g per day.

Based on the protocol developed by the European Food Safety Authority (EFSA) [16], energy misreporting was assessed as the ratio of reported energy intake (EI) to estimated basal metabolic rate (EI:BMR) according to the Goldberg method [17] modified by Black [18]. The FFQs were used to estimate reported EI and BMR was calculated using the validated sex- and age-specific Oxford equations suitable for use in populations with a range of weight statuses [19]. A moderately-active physical activity level (PAL) of 1.6 was assumed for all participants [20]. Misreporters of dietary intake were identified by EI:BMR ratios <0.901 (underreporters) or >2.841 (overreporters). Fifty-eight participants were classified as misreporters (17 females and 41 males), and further statistical analysis was performed on a total of 157 subjects (77 females, aged 23–77 years, and 80 males, aged 25–76 years).

#### 2.2.2. Comparison with Dietary Reference Values

In each subject, adequacy was assessed by comparing energy and nutrient intakes with age-/sex-specific EFSA dietary reference values (DRVs) [21] or nutrient requirements and dietary guidelines of WHO/FAO [22,23] if the former were not available. Specifically, the following daily intakes were considered adequate:

- Energy ranging between EFSA DRVs for energy calculated according to age using PAL values of 1.4 and 1.8, which approximately reflect low active (sedentary) and active lifestyles (6.8–10.1 MJ/day and 8.3–12.6 MJ/day ranges for females and males, respectively);
- Total carbohydrates ranging from 45 to 60% energy (%EI);
- Sugars (monosaccharides and disaccharides) <10%EI based on the WHO/FAO dietary recommendations;
- Dietary fibre intake ≥25 g/day;
- Protein between the average requirement (AR) and the population reference intake (PRI) of EFSA DRVs;
- Total fat ranging from 20 to 35%EI;
- Saturated fatty acid (SFAs) <10%EI according to FAO;
- Total unsaturated fatty acids (UFAs), i.e., monounsaturated fatty acids (MUFAs) plus polyunsaturated fatty acids (PUFAs) ranging from 15 to 20%EI, as calculated by difference according to FAO;
- PUFAs ranging from 6 to 11%EI according to FAO.

In addition, a moderate alcohol consumption (up to one serving per day for women and up to two servings per day for men) [24] was considered acceptable.

Differences between current intake and corresponding recommended intake (mean value of recommended range for energy, total carbohydrates, protein, total fat, and total UFAs and PUFAs; limit of adequate intake for sugars, dietary fibre, SFAs, and alcohol) were calculated. The resulting delta values were then used for elaborating on the predictive model.

### 2.3. Statistical Analysis

Data were stratified by gender. All clinical parameters were classified as normal (1) or abnormal (2) according to their overlap with the recognised normal ranges (Table S2). The distribution of clinical parameters was evaluated using the D’Agostino–Pearson test. Student’s t-test for normally distributed data and Mann–Whitney U test for non-normally distributed data were used to compare the general characteristics of the study population grouped by gender.

All statistical analyses were conducted using the Python programming language, using custom scripts and the *sklearn* package [25]. A predictive model for each clinical parameter was computed using all dietary variables via a ridge regression framework [26].

To simultaneously reach the best prediction performances while learning which sets of dietary macronutrient intakes (variables) were the most important for each predicted clinical outcome (target), a multivariate model was applied. To this end, a model selection was performed in order to find the best regression model. Since no univariate effect of a single nutritional variable on the clinical targets emerged (data not shown), we assumed that multivariate techniques were the most promising methods as they are capable of simultaneously reaching the best prediction performances while learning which sets of variables are the most important for each prediction task. Indeed, penalised maximum likelihood methods (LASSO regression, ridge regression) outperformed other classes of regression models as previously shown in other nutritional studies [27,28]. In particular, the ridge regression yielded the best fit on the data under study. The ridge regression belongs to the wider class of penalised linear regressions. These types of models allow computing a regression while shrinking the coefficients of uninformative variables. The linear ridge regression minimises the function:

(1) $$\overset{n}{\underset{i=1}{\sum }}(y_{i}-\beta_{0}-\overset{p}{\underset{j=1}{\sum }}\beta_{j}x_{ij})²+\alpha \overset{p}{\underset{j=1}{\sum }}\beta_{j}^{2}=RSS+\alpha \overset{p}{\underset{j=1}{\sum }}\beta_{j}^{2}$$

*RSS* = residual sum of squares, with *i* = index of summation for observations, *n* = number of observations (1 to 77 for women, 1 to 80 for men), *j* = index of summation for variables, *p* = number of variables (1 to 10).

The penalty coefficient α can be tuned to optimise the bias-variance trade-off of the model, leading to a maximisation of the predictive performance as a function of the smallest set of the descriptive variables necessary to achieve said performance. The penalty term introduced by the ridge regression is useful to deal with multicollinearity and prevent overfitting; for the present case, it translated to the shrinkage of coefficients of dietary variables strongly correlated among themselves and weakly correlated to a given clinical marker. The absolute value of the regression coefficients β is related to the univariate effect of a given dietary variable (*x*)*_j_* on a given clinical marker (*y*), while the sign of coefficients is not directly interpretable as it would have been in a normal ordinary least squares (OLS) solution.

Before regression, data were standard scaled. All the ridge models computed were cross-validated to optimise the parameter α through 5-fold cross-validation. Train and test subsets were extracted to maintain the proportion between recruiting centres to minimise the possible confusion factor tied to dietary habits of the country of origin.

To represent the statistical dependence between the rankings of dietary variables and clinical outcomes, correlation heatmaps were also computed using the Spearman rank correlation coefficient [29], which measures how well the relationship between two variables or targets can be described using a monotonic function. The Spearman rank correlation coefficient allows for nonlinear relationships to be detected, providing a good description of the relationships between features and targets.

## 3. Results and Discussion

Table 1 summarises the characteristics of volunteers included in the study. As expected, a significant heterogeneity was evidenced between men and women, possibly due to different hormonal profiles and body fat distribution [30,31].

Ridge-type penalisation was obtained retaining all predictors and minimising collinearity amongst variables; it performed better than LASSO probably due to the complexity of interactions of all the dietary variables in defining the overall clinical picture in the subjects at risk of MetS. Indeed, ridge regression performs better when many predictors have coefficients of roughly equal size [32]. The Pearson correlation coefficients of determination (R^2^) for the clinical outcomes according to the ridge regression are reported in Table 2.

**Figure 1:**
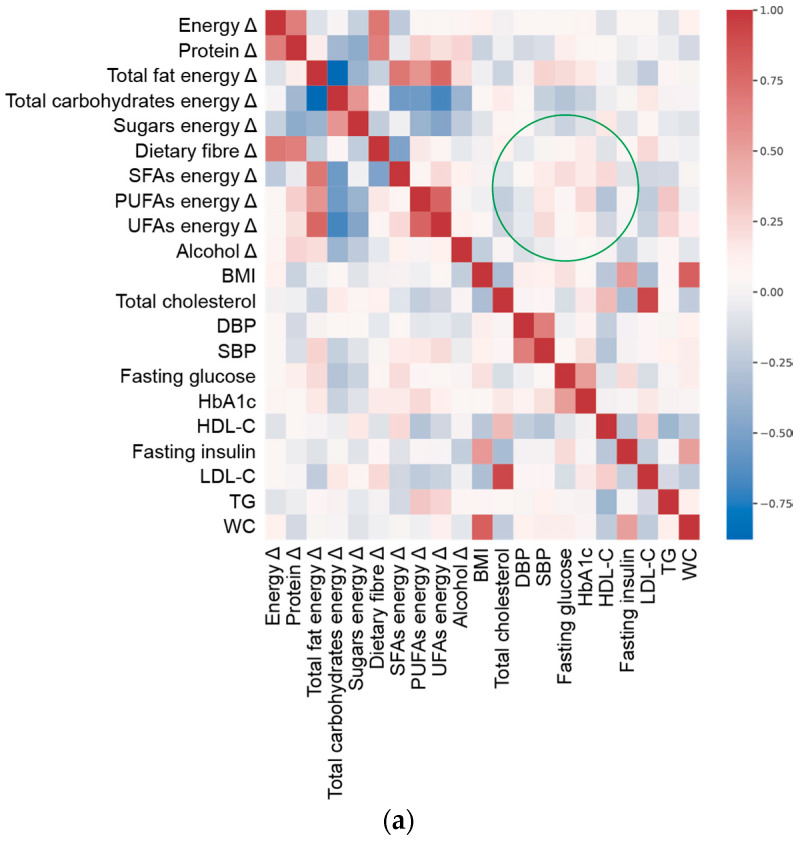

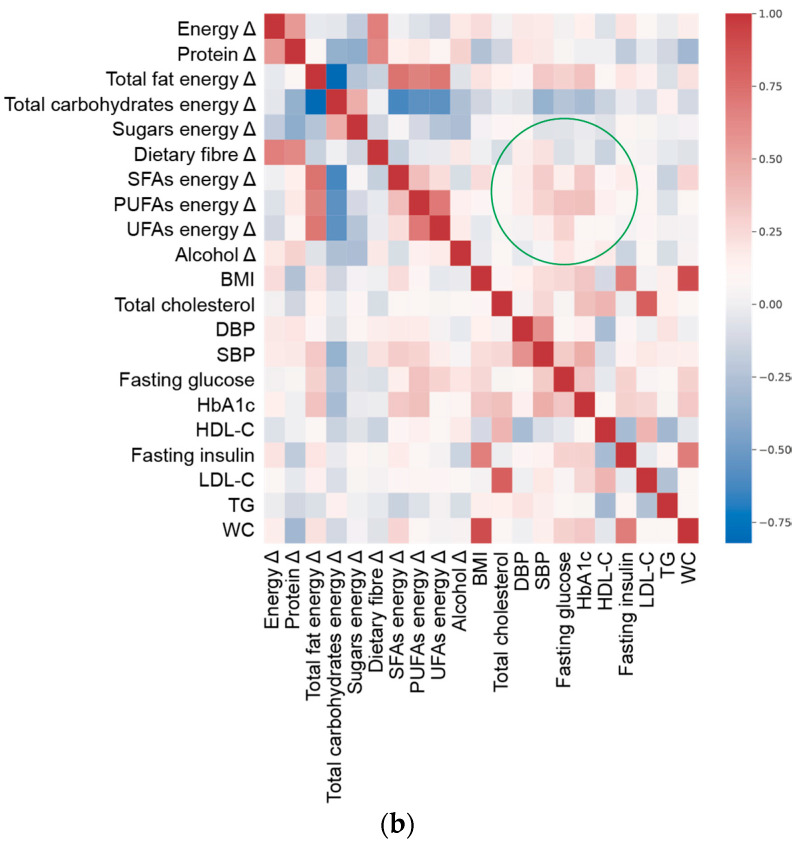
Spearman’s correlation heat map of adequacy of energy/macronutrient intake (Δ, delta value calculated as difference between current intake and corresponding recommended intake of each variable) and clinical parameters at enrolment (Table 1) in (**a**) females and (**b**) males. Green circle marks an example of a different pattern in correlation between variables and targets in males and females.

To visualize the overall complexity of the relationships between clinical parameters and nutritional variables, we computed the heatmap of correlations between them (Figure 1). The topology of the heatmaps for male and female study participants was slightly different, highlighting the gender-related differences in clinical and nutritional characteristics associated with MetS. Within these premises, the linear ridge regression has been chosen as the best trade-off between predictive performances and interpretability of results.

Based on the results of the predictive model (Table 2 and Figure 1), we focused on clinical features that were better estimated by the adequacy/inadequacy of dietary intakes (R^2^ > 0.4, as a generally accepted standard [33]), highlighting the variables characterising the prediction. To this aim, ridge regression performance (Figure S1) and the magnitude of regression coefficients (Figure S2) were plotted per individual clinical outcome.

According to the ridge regression results, inadequate dietary intakes better predicted BMI in males (R = 0.78) than in females (R = 0.43). In both genders, exceeding energy intake was the key predictor of a high BMI, especially for obese subjects, confirming that BW changes are associated with an imbalance between energy intake and expenditure [23]. Based on our predictive model, overweight and obesity were also predicted by elevated intake of total fat (>35%EI) in males and of SFAs (>10%EI) in both genders. This is in accordance with outcomes from epidemiologic studies and clinical trials, which suggest that total fat [34] and SFA intake [35] are strongly linked to BW. Excessive intake of SFAs was also a characterising predictor variable of high WC, which was well estimated by inadequate dietary intakes in males (R = 0.79). In males, low consumption of protein was a good indicator of both elevated BMI and WC.

Our results suggest that consumption of high-energy, low-protein, and high-fat diets, particularly when including excessive SFAs, strongly relates to the development of obesity in men and to a lesser extent in women. This gender-related difference confirms results from long-term prospective studies evidencing a significant positive connection between weight gain and dietary fat in a cohort including males and females [36], while energy content from fat was weakly correlated with weight gain in The Nurses’ Health Study, including only women [37].

Interestingly, neither total carbohydrate nor sugar intake were predictors of overweight/obesity. Epidemiological evidence and results from diet intervention trials suggest that protein and carbohydrate intakes are inversely related to BMI, while excessive intake of sugars contributes to obesity [38]. Although the plausibility of the mechanisms provides support for a role of sugar consumption in the epidemics of overweight/obesity, definitive studies are missing [39]. In our group of subjects at risk of MetS, the predictivity of sugar intake on BMI was low and supported the conclusion that there is no clear or convincing evidence that any dietary or added sugars have a unique or detrimental impact relative to any other source of calories on the development of obesity [40].

Results from the ridge regression indicated that blood TG were better predicted by the dietary variables in women (R = 0.46) than in men (R = 0.22). In the female subjects, correct (6–11%E) or slightly low PUFA intake and correct fibre intake (25 g/day) had a good predictive value of normal TG level, confirming the importance of dietary fibre in the maintenance of normal blood TG [41]. Although the total PUFA intake must be considered first when examining dietary habits affecting lipemia [42], it is documented that high n-3 PUFA intake favourably impacts on blood TG [43], while excessive consumption of n-6 PUFAs may lead to negative effects [44]. In this study, it was not possible to accurately discriminate between n-6 and n-3 PUFA intake and it could explain why high PUFA intake did not predict normal TG level.

In females, inadequate dietary intakes, mainly high consumption of available carbohydrates and fats, predicted high LDL-C, and adequate total PUFA intake was a predictive variable of normal LDL-C (R = 0.42). In women, low HDL-C was well predicted by inadequate intakes (R = 0.44), mainly excessive energy, SFA, and available carbohydrates. Although the magnitude scale of the ridge coefficient was generally low for HDL-C prediction (Figure S2), denoting a prediction characterised by the combined effect of nutrients rather than a single dietary variable, overall, our data confirmed evidence in the literature about the negative effect of excessive carbohydrate intake on dyslipidaemia [45,46]. Total cholesterol was not well predicted by any dietary intake both in females (R = 0.35) and males (R = 0.25).

Clinical features related to glucose metabolism and insulin sensitivity were better predicted in men than in women. In males, fasting glucose was well predicted (R = 0.44), and elevated total fat and SFA intakes were slightly associated with moderate fasting hyperglycaemia. These results are consistent with evidence demonstrating that excessive consumption of total fat [47] and SFAs [48,49] favour the onset of insulin resistance. Although no univariate effect of any specific dietary variable was evidenced, fasting insulin and glycated haemoglobin (HbA1c) were moderately predicted by the combined effect of all dietary nutrients in the male group (R = 0.49 and R = 0.52, respectively).

DBP was not well predicted by the examined dietary variables either in women (R = 0.27) or men (R = 0.40). Conversely, inadequate dietary intakes well predicted high SBP in males (R = 0.52), and excessive total fat intake concomitant to low PUFA intake was a good predictor of moderate hypertension (140–159 mmHg) (Figure S1). Again, our results confirmed evidence from observational and epidemiological studies [50,51,52].

Although several studies found an association between alcohol drinking and the prevalence of MetS and most of its components, in our study, alcohol consumption was not predictive of any clinical outcome. We speculate that this was related to the very low percentage of enrolled volunteers exceeding the acceptable consumption of alcoholic beverages (6.5% of females and 16.5% of males), which did not allow any stratification based on alcohol intake.

In summary, in the herein reported retrospective analysis, we focused on the predictive effect of energy/macronutrient intake on the clinical features related to the risk of MetS. We did not focus on food intake and/or dietary pattern, of which their contribution to the risk of MetS has already been addressed by several studies (see [53] for a comprehensive review). Although this approach has limitations since components other than energy and macronutrients are provided by food/diet, our results suggest that predictivity of inadequate intake of energy/macronutrients is independent of dietary patterns. Indeed, we evaluated four different cohorts with different dietary habits tied to the geographical origins of the volunteers, and train and test batches of the cross-validation were stratified with respect to the nationality of each subject to avoid biases derived from different eating habits.

Overall, energy/macronutrient intake had a strong predictivity. We speculate that this relies on the intimate relationship between MetS and obesity, which is in turn strongly dependent on the unbalance of energy/macronutrients in the diet. Of note, not all clinical outcomes were predicted with the same accuracy, and the predictivity was overall higher in men than in women. Furthermore, inadequate intake of specific nutrients was associated to abnormality of specific clinical parameters. Most of the observed intake/clinical outcome associations were consistent with previous evidence. This does not mean that our results are trivial and simply confirmatory, but rather it confirms that the proposed model is suitable for shedding light on the complexity of nutritional variables that, although responsible for impacting on clinical outcomes and, therefore, for influencing the pathological condition, have an effect that is not evident with univariate analysis and must be considered in the framework of the reciprocal influence of the other variables.

The impact of physical activity and smoking was not considered in our model, and this is a limitation since they are both included among lifestyle factors predictive for MetS [54]. Of note, none of the enrolled volunteers was a heavy smoker (≥5 cigarettes per day); this minimising the confounding effect and making a stratification based on these lifestyle characteristics impossible. As well, based on exclusion criteria, none of enrolled subjects had a high level of physical activity (≥5 h of physical activity per week). Specific information on physical activity was collected using the international physical activity questionnaire (IPAQ) only for volunteers who accepted to participate in the sub-study of the trial, so we could not use those data in the regression model. Anyway, collected IPAQ confirmed that physical activity was low-moderate.

## 4. Conclusions

In this retrospective study, the energy and macronutrient intake of 157 (80 males and 77 females) adult volunteers at risk of MetS from four different countries was evaluated using a validated standardised protocol to measure dietary intakes. The use of the ridge regression, which optimises prediction performances while retaining information about the role of all the nutritional variables, allowed us to assess if a clinical outcome is strongly dependent on a single nutritional variable, or if its prediction is characterised by more complex interactions between the variables. The approach appeared robust, and although our results cannot be applied to the general population, they allowed for the linking of energy/macronutrient intake to the clinical features of MetS, thus providing additional indications about the most important dietary contributors to the risk of the disease.

Methods in prediction modelling have been recently growing and are becoming more relevant in the nutrition field [55]. In the near future, they could be useful to healthcare professionals and policymakers to effectively counteract the risk of MetS and other diet-related diseases.

## Figures and Tables

**Table 1:** General characteristics of the study population grouped by gender (medians and interquartile ranges, IQR).

	Women	Men	
	Median (IQR) ^†^	Median (IQR) ^†^	*p* ^‡^
Subjects (n; %)	77; 49.0%	80; 51.0%	–
Age (years)	58 (50–66)	54 (46–63)	0.0631
BMI (kg/m^2^)	31.6 (27.5–35.5)	29.0 (26.1–33.0)	0.0187
WC (cm)	100.5 (93.0–111.0)	104.5 (99.0–113.5)	0.0489
TG (mg/dL)	160.5 (125.1–192.6)	188.9 (153.1–239.1)	0.0006
Total cholesterol (mg/dL)	232.6 (201.4–254.2)	202.7 (188.6–230.1)	<0.0001
HDL-C (mg/dL)	48.2 (41.7–56.6)	39.2 (35.2–42.6)	<0.0001
LDL-C (mg/dL)	164.5 (138.0–180.7)	136.0 (112.2–154.8)	<0.0001
Fasting glucose (mg/dL)	96.1 (87.4–100.8)	97.0 (89.2–102.6)	0.3454
Fasting insulin (µIU/mL)	12.4 (7.9–18.4)	12.9 (9.7–18.5)	0.5382
HbA1c (%)	5.6 (5.3–5.8)	5.3 (5.1–5.6)	0.0022
SBP (mmHg)	130.0 (120.0–145.0)	130.0 (125.0–141.5)	0.2527
DBP (mmHg)	81.0 (76.0–89.0)	85.0 (80.0–91.0)	0.0057

Abbreviations: BMI: body mass index; DBP: diastolic blood pressure; HbA1c: glycated haemoglobin; HDL-C: high-density lipoprotein (HDL) cholesterol; IQR: interquartile ranges; IU: international units; LDL-C: low-density lipoprotein (LDL) cholesterol; SBP: systolic blood pressure; TG: triglycerides; WC: waist circumference. ^†^ Median (IQR) for all parameters, except subjects (n; %). ^‡^ *p* values from Student’s *t*-test for normally distributed variables (WC, total cholesterol, LDL-C, DBP) and Mann–Whitney U test for non-normally distributed variables (age, BMI, TG, HDL-C, fasting glucose, fasting insulin, HbA1c, SBP).

**Table 2:** Pearson correlation coefficients of determination (R^2^) for the clinical outcomes according to the ridge regression. R^2^ > 0.4 are in bold.

	Women	Men
	R^2 †^	R^2 †^
BMI	**0.43**	**0.78**
WC	0.39	**0.79**
TG	**0.46**	0.22
Total cholesterol	0.35	0.25
HDL-C	**0.44**	0.34
LDL-C	**0.42**	0.22
Fasting glucose	0.33	**0.44**
Fasting insulin	0.26	**0.49**
HbA1c	0.27	**0.52**
SBP	0.36	**0.52**
DBP	0.27	0.40

^†^ The Pearson correlation coefficient of determination (R^2^) of each clinical outcome is the average of the results of each validation fold.

## Data Availability

The data presented in this study are available on request from the corresponding author.

## Supplementary Materials

The following are available online at https://www.mdpi.com/article/10.3390/nu13041377/s1, Table S1: Exclusion criteria of the study, Table S2: Cut-off target ranges of the clinical and metabolic parameters, Figure S1: Scatterplots of ridge regression performance per clinical outcome in females and males, Figure S2: Magnitude of regression coefficients.

## Availability of Supporting Data

The Python source code for analysis and plotting is available at https://github.com/CarloMengucci/Model-selection-and-learning-for-nutritional-data (accessed on 15 April 2021).
