# Supplementary material for: Unveiling the Correlation between Inadequate Energy/Macronutrient Intake and Clinical Alterations in Volunteers at Risk of Metabolic Syndrome by a Predictive Model
Source: Nutrients. 2021 Apr 20;13(4):1377. doi: 10.3390/nu13041377 (PMC8072695; doi:10.3390/nu13041377)
Supplement: Supplementary file 1 [file nutrients-13-01377-s001.zip › Danesi et al_supplementary data.pdf]

SUPPLEMENTARY DATA

SUPPLEMENTARY TABLES

Table S1. Exclusion criteria of the study.

**Exclusion criteria**
– Participants meeting all five criteria for metabolic syndrome (MetS);
– Diagnosis of heart, liver, or kidney failure (other organ diseases were assessed case-by-case by the study doctor);
– Regular drug therapy with impact on serum lipids (e.g., statins, fibrates);
– Diabetes (fasting glucose >126 mg/dL, or anti-diabetic treatment);
– Overt hypothyroidism (subjects with ongoing thyroxine treatment and normal TSH levels could be included);
– Familial dyslipidaemia;
– Recent history of cancer or cancer treatment (less than two years);
– Active or recently diagnosed intestinal malabsorption or disorders associated with malabsorption: Crohn's disease, short bowel syndrome, pancreatic insufficiency, cystic fibrosis, Tropical Sprue, Whipple's disease, chronic pancreatitis, gastrojejunostomy, surgical treatments for obesity, cholestasis, biliary atresia, parasite infections, HIV/AIDS;
– Use of medication known to cause malabsorption: tetracycline, cholestyramine, aluminium/magnesium hydroxide, colchicine, neomycin, methotrexate, methyldopa, allopurinol, and laxatives;
– Antibiotic treatment within the last month;
– Illegal drug use, chronic alcoholism;
– Smoking ≥5 cigarettes per day;
– Intensive physical exercise (≥5 hours per week);
– History of allergy or intolerance to any components used in bioactive-enriched foods (BEF), celiac disease, lactose intolerance, allergy to milk or egg proteins;
– Consumption of nutritional supplements containing docosahexaenoic acid (DHA), β-glucans (BG), or anthocyanins (AC);
– Women who are pregnant, lactating or actively trying to conceive;
– Institutionalised patients, those who lack the autonomy to consent or are unable to meet all examinations;
– Participation in other clinical trials that may impact on outcome;
– Subjects deprived of their liberty by judicial or administrative decision.

Table S2. Cut-off target ranges of the clinical and metabolic parameters.

Clinical and metabolic parameters		Cut-off target ranges	Reference
**Anthropometric characteristics**	**Body mass index (BMI)**		
	Normal	18.5–24.9 kg/m^2	[1]
	High	≥25 kg/m^2	
	**Waist circumference (WC)**		
	Normal	<88 cm for women, <102 cm for men	[2]
	High	≥88 cm for women, ≥102 cm for men	
**Lipid profile**	**Triglycerides (TG)**		
	Normal	<150 mg/dL	[2]
	High	≥150 mg/dL	
	**Total cholesterol**		
	Normal	<200 mg/dL	[2]
	High	≥200 mg/dL	
	**LDL cholesterol (LDL-C)**		
	Normal	<160 mg/dL	[2]
	High	≥160 mg/dL	
	**HDL cholesterol (HDL-C)**		
	Normal	women >50 mg/dL, men >40 mg/dL	[2]
	Low	women ≤50 mg/dL, men ≤40 mg/dL	
**Glucose profile**	**Fasting glucose**		
	Normal	<100 mg/dL	[3]
	High	≥100 mg/dL	
	**Fasting insulin**		
	Normal	≤25 μIU/mL	[4]
	High	>25 μIU/mL	
	**Glycated haemoglobin (HbA1c)**		
	Normal	≤6%	[3]
	High	>6%	
**Haemodynamic variables**	**Systolic blood pressure (SBP)**		
	Normal	<140 mmHg	[5]
	High	≥140 mmHg	
	**Diastolic blood pressure (DBP)**		
	Normal	<90 mmHg	[5]
	High	≥90 mmHg	

SUPPLEMENTARY FIGURES

Figure S1. Scatterplots of ridge regression performance per clinical outcome in (**a**) females and (**b**) males. Each plot is coloured with a continuous gradient related to the position of each subject (dot) in the distribution of each dietary variable. The gradient is computed on normalised values of the intake of each variable so that an extremely scarce intake gets the value –1 (indigo) and an extremely excessive intake gets the value 1 (dark red). Values within the recommended range are normalised accordingly, with the spectre of yellow denoting a correct intake (delta ≈ 0). Only clinical outcomes with R^2>0.4 according to the ridge regression are reported.

(**a**) Women

Body mass index (BMI)

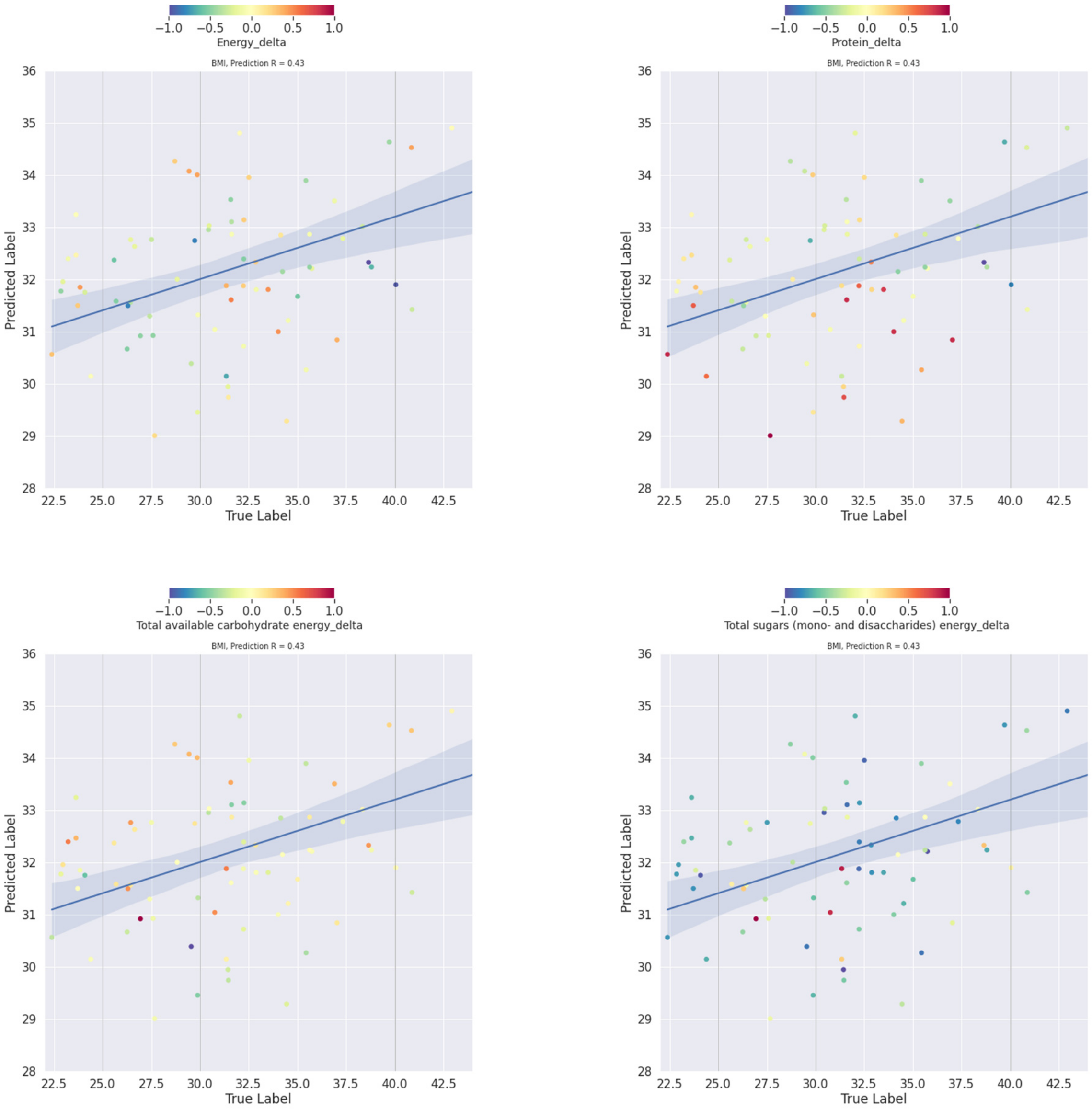

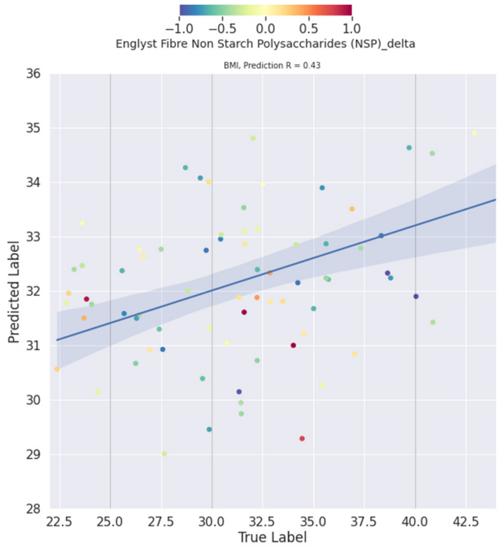
−1.0 −0.5 0.0 0.5 1.0
Englyst Fibre Non Starch Polysaccharides (NSP)_delta
BMI, Prediction R = 0.43
Predicted Label
True Label

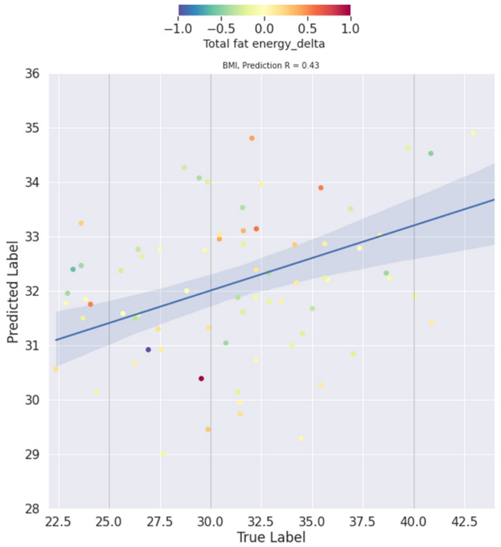
−1.0 −0.5 0.0 0.5 1.0
Total fat energy_delta
BMI, Prediction R = 0.43
Predicted Label
True Label

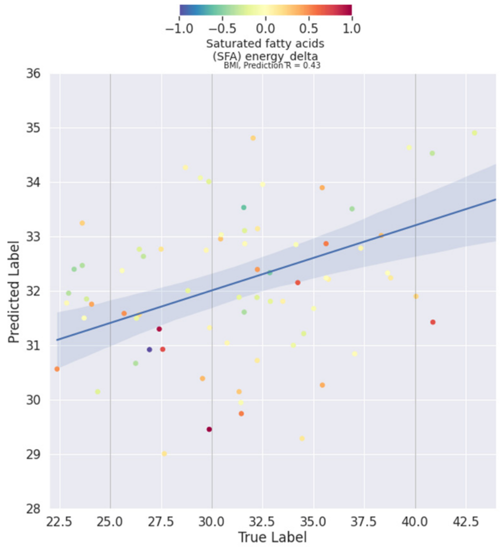
−1.0 −0.5 0.0 0.5 1.0
Saturated fatty acids
(SFA) energy_delta
BMI, Prediction R = 0.43
Predicted Label
True Label

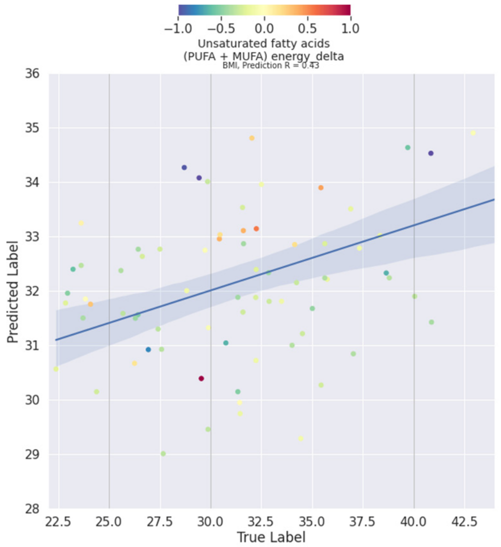
−1.0 −0.5 0.0 0.5 1.0
Unsaturated fatty acids
(PUFA + MUFA) energy_delta
BMI, Prediction R = 0.43
Predicted Label
True Label

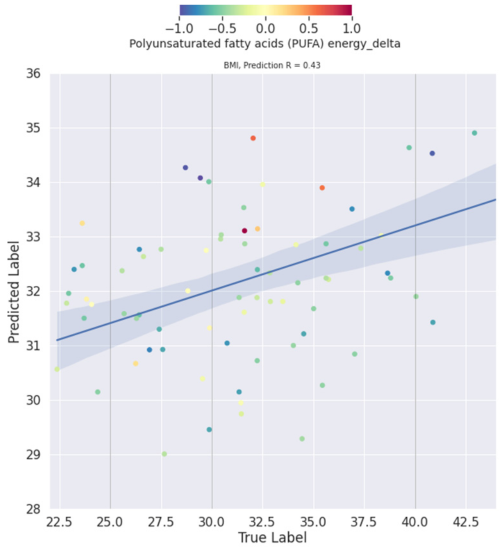
−1.0 −0.5 0.0 0.5 1.0
Polyunsaturated fatty acids (PUFA) energy_delta
BMI, Prediction R = 0.43
Predicted Label
True Label

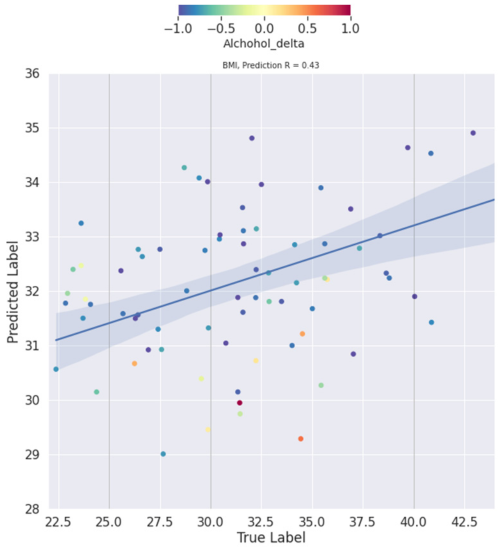
−1.0 −0.5 0.0 0.5 1.0
Alchohol_delta
BMI, Prediction R = 0.43
Predicted Label
True Label

Triglycerides (TG)

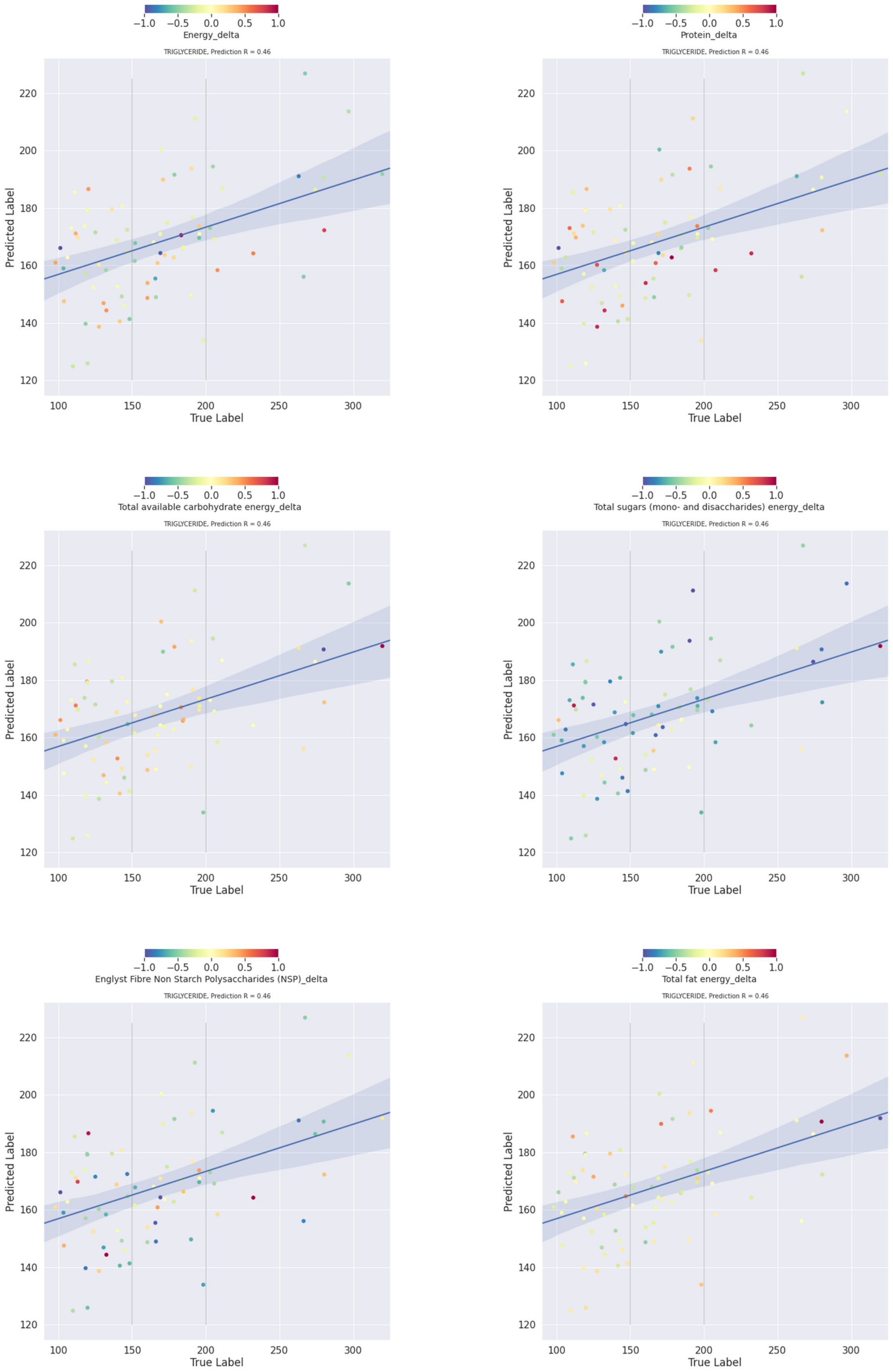

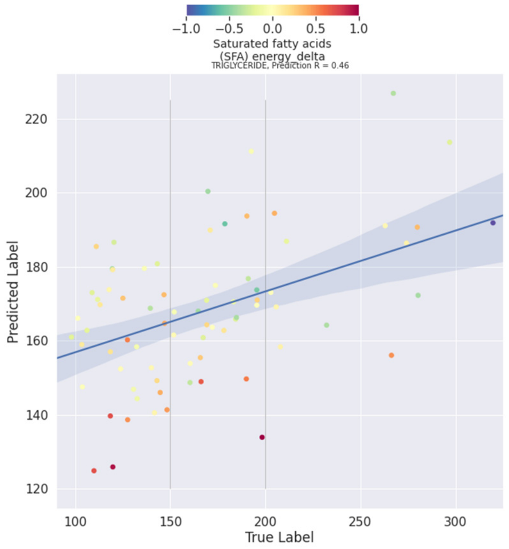

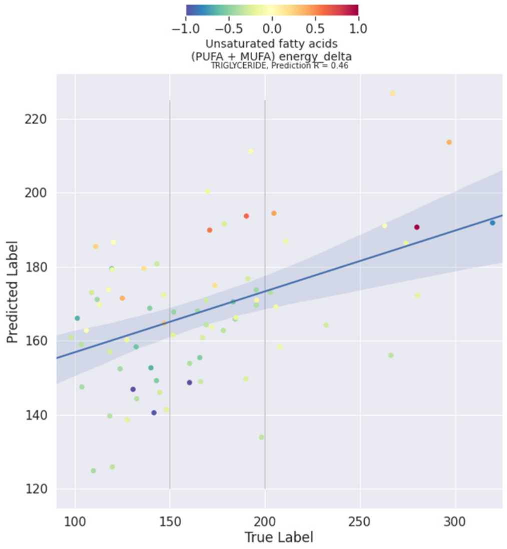

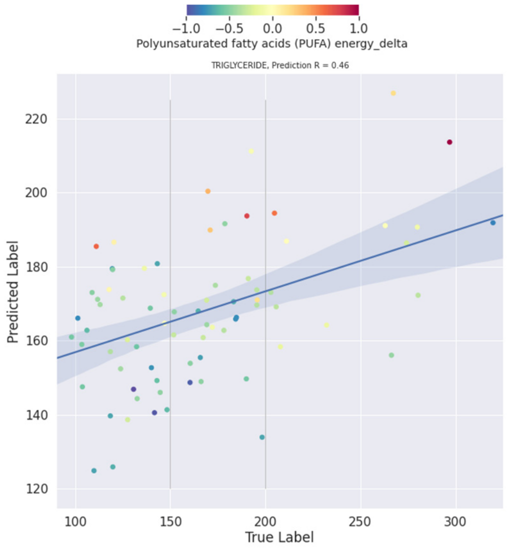

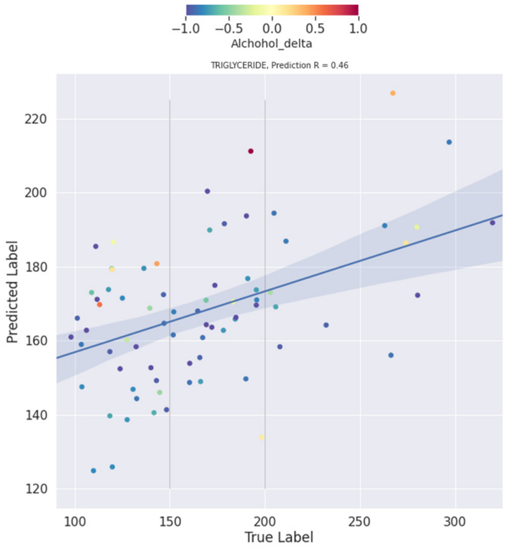

LDL cholesterol (LDL-C)

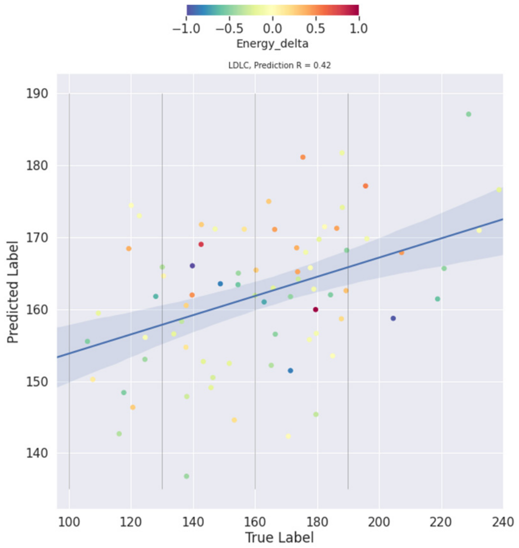

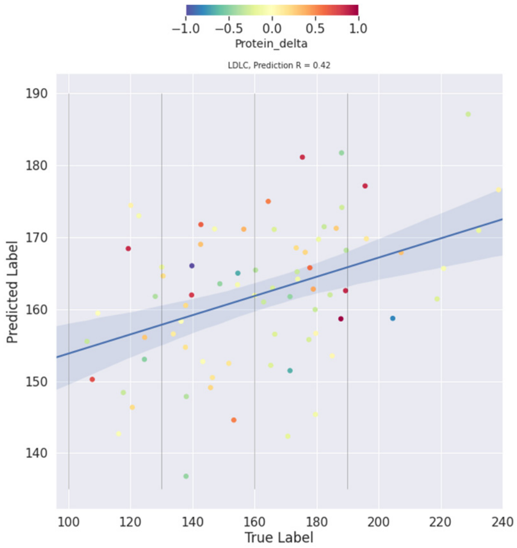

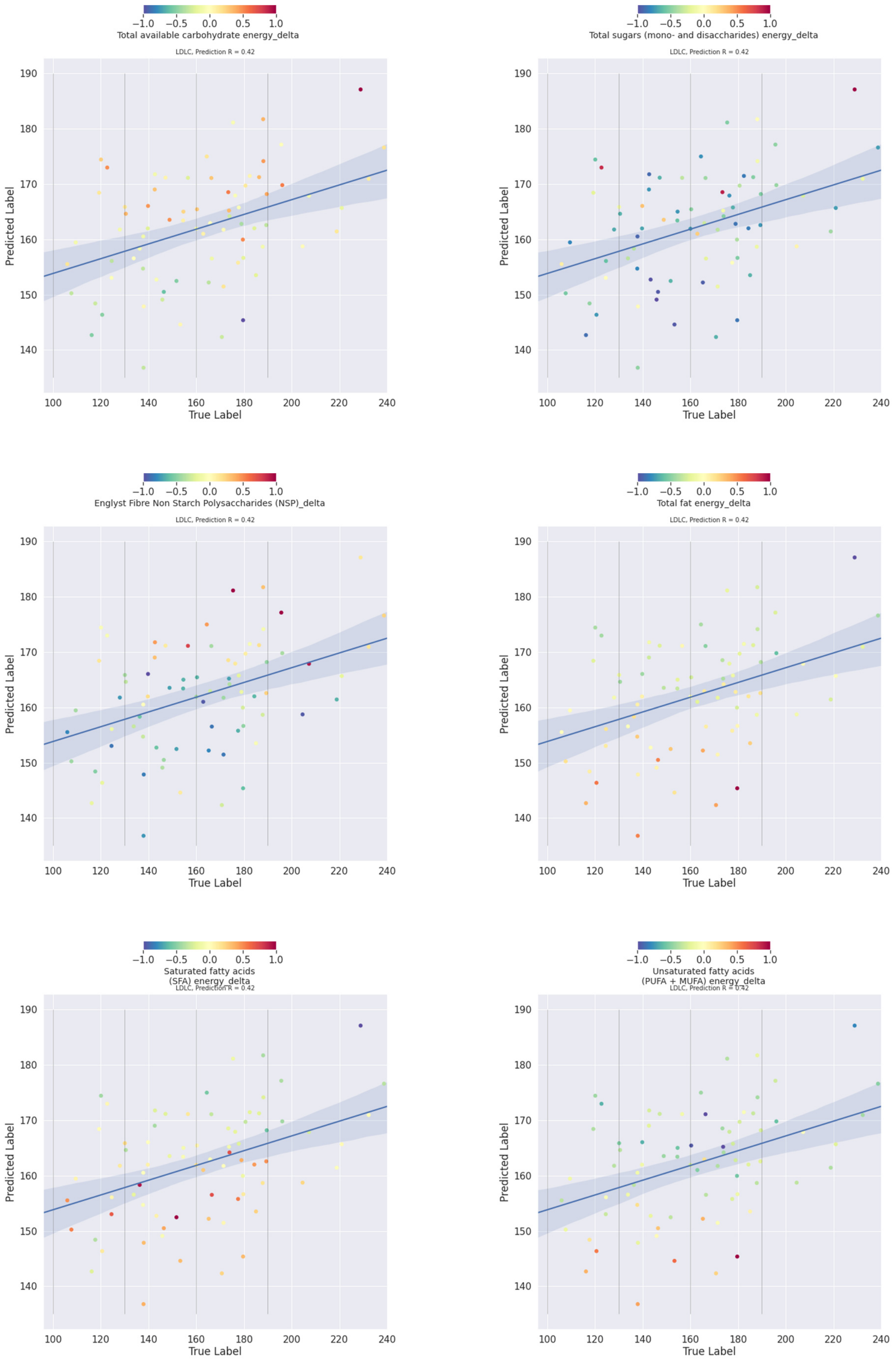
−1.0 −0.5 0.0 0.5 1.0
Total available carbohydrate energy_delta
LDLC, Prediction R = 0.42
−1.0 −0.5 0.0 0.5 1.0
Total sugars (mono- and disaccharides) energy_delta
LDLC, Prediction R = 0.42
−1.0 −0.5 0.0 0.5 1.0
Englyst Fibre Non Starch Polysaccharides (NSP)_delta
LDLC, Prediction R = 0.42
−1.0 −0.5 0.0 0.5 1.0
Total fat energy_delta
LDLC, Prediction R = 0.42
−1.0 −0.5 0.0 0.5 1.0
Saturated fatty acids
(SFA) energy_delta
LDLC, Prediction R = 0.42
−1.0 −0.5 0.0 0.5 1.0
Unsaturated fatty acids
(PUFA + MUFA) energy_delta
LDLC, Prediction R = 0.42
Predicted Label
True Label
140 150 160 170 180 190
100 120 140 160 180 200 220 240

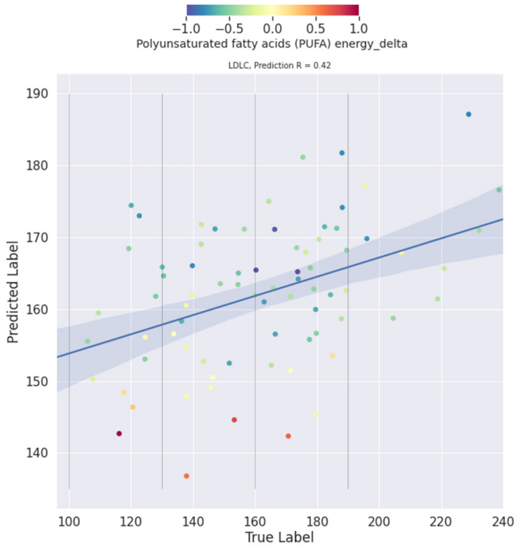

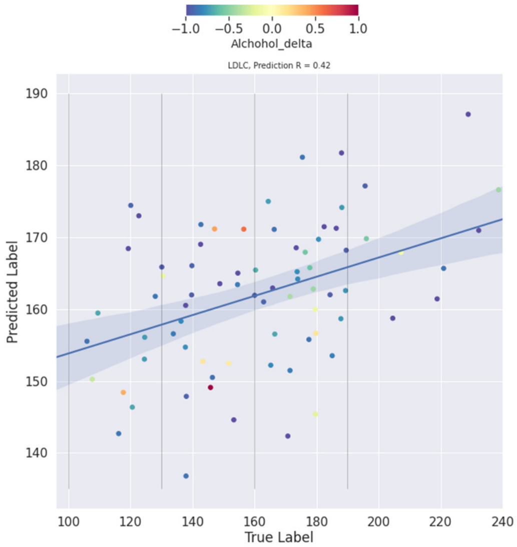

HDL cholesterol (HDL-C)

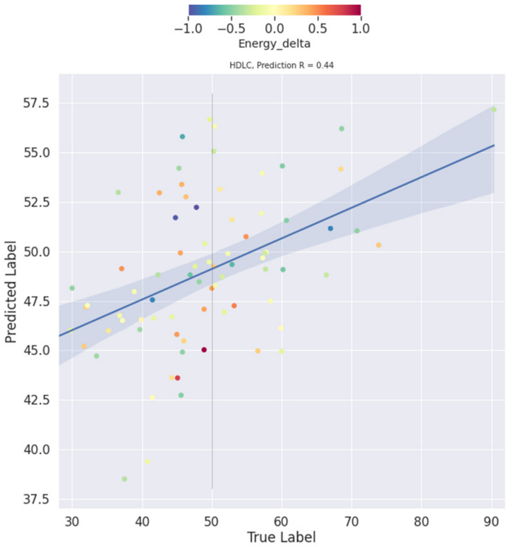

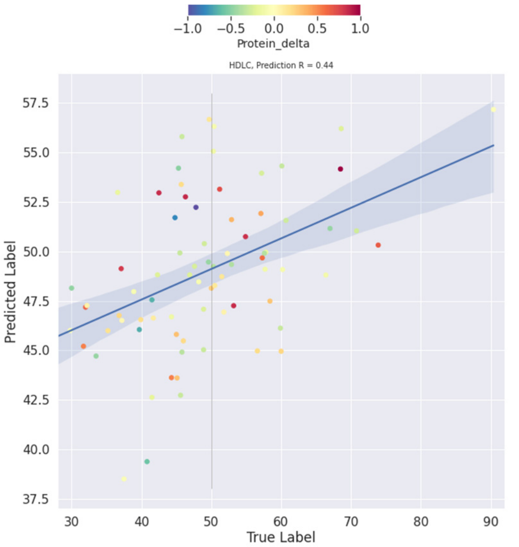

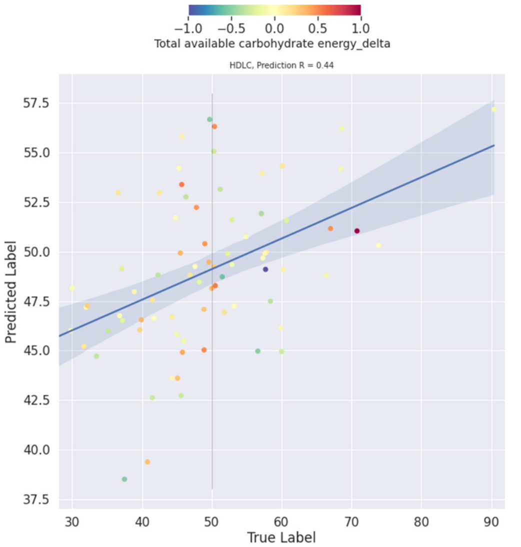

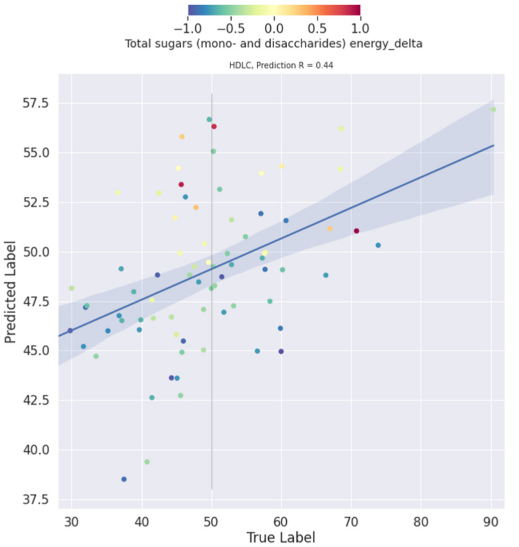

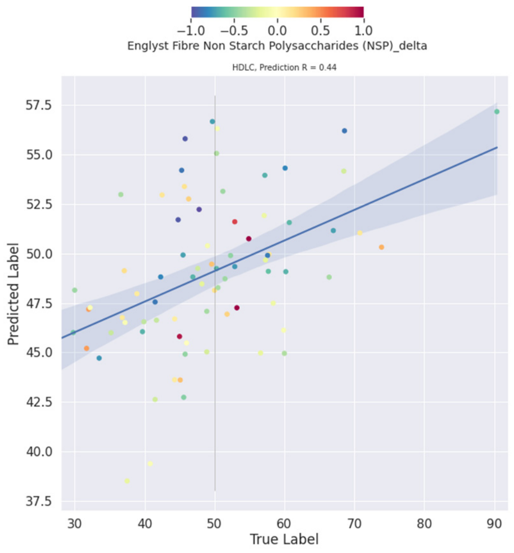
Englyst Fibre Non Starch Polysaccharides (NSP)_delta
HDLC, Prediction R = 0.44
Predicted Label
True Label

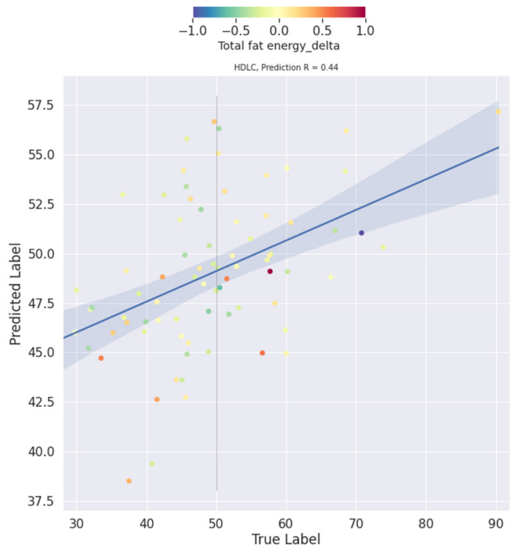
Total fat energy_delta
HDLC, Prediction R = 0.44
Predicted Label
True Label

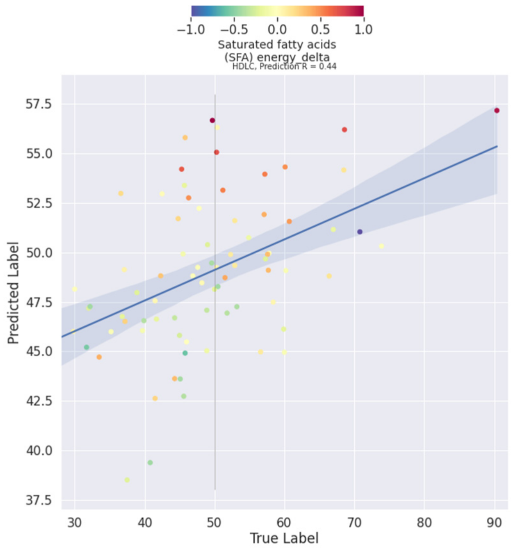
Saturated fatty acids
(SFA) energy_delta
HDLC, Prediction R = 0.44
Predicted Label
True Label

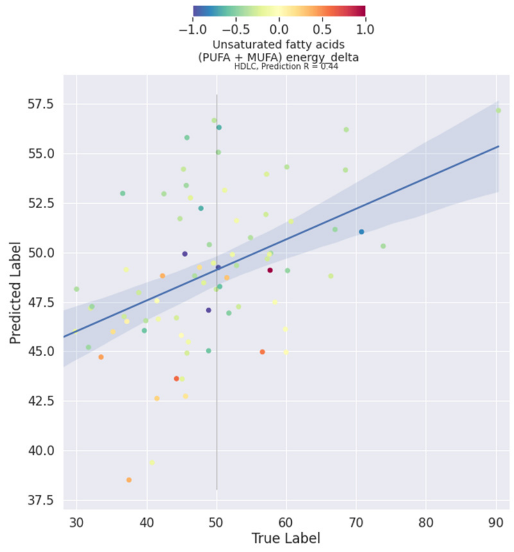
Unsaturated fatty acids
(PUFA + MUFA) energy_delta
HDLC, Prediction R = 0.44
Predicted Label
True Label

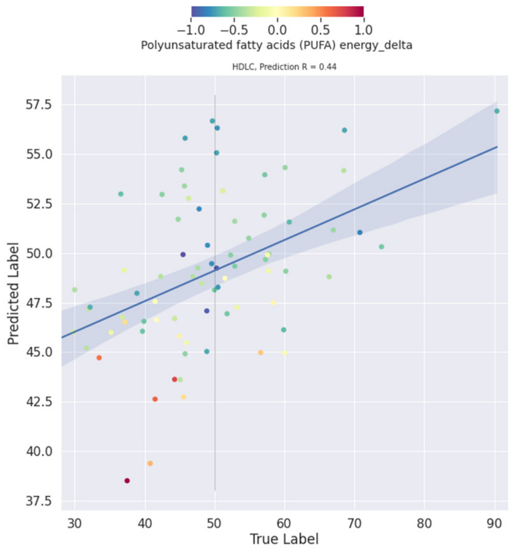
Polyunsaturated fatty acids (PUFA) energy_delta
HDLC, Prediction R = 0.44
Predicted Label
True Label

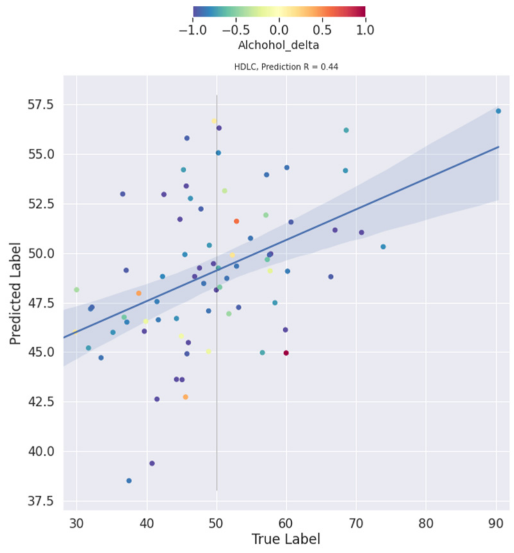
Alchohol_delta
HDLC, Prediction R = 0.44
Predicted Label
True Label

(**b**) Men

Body mass index (BMI)

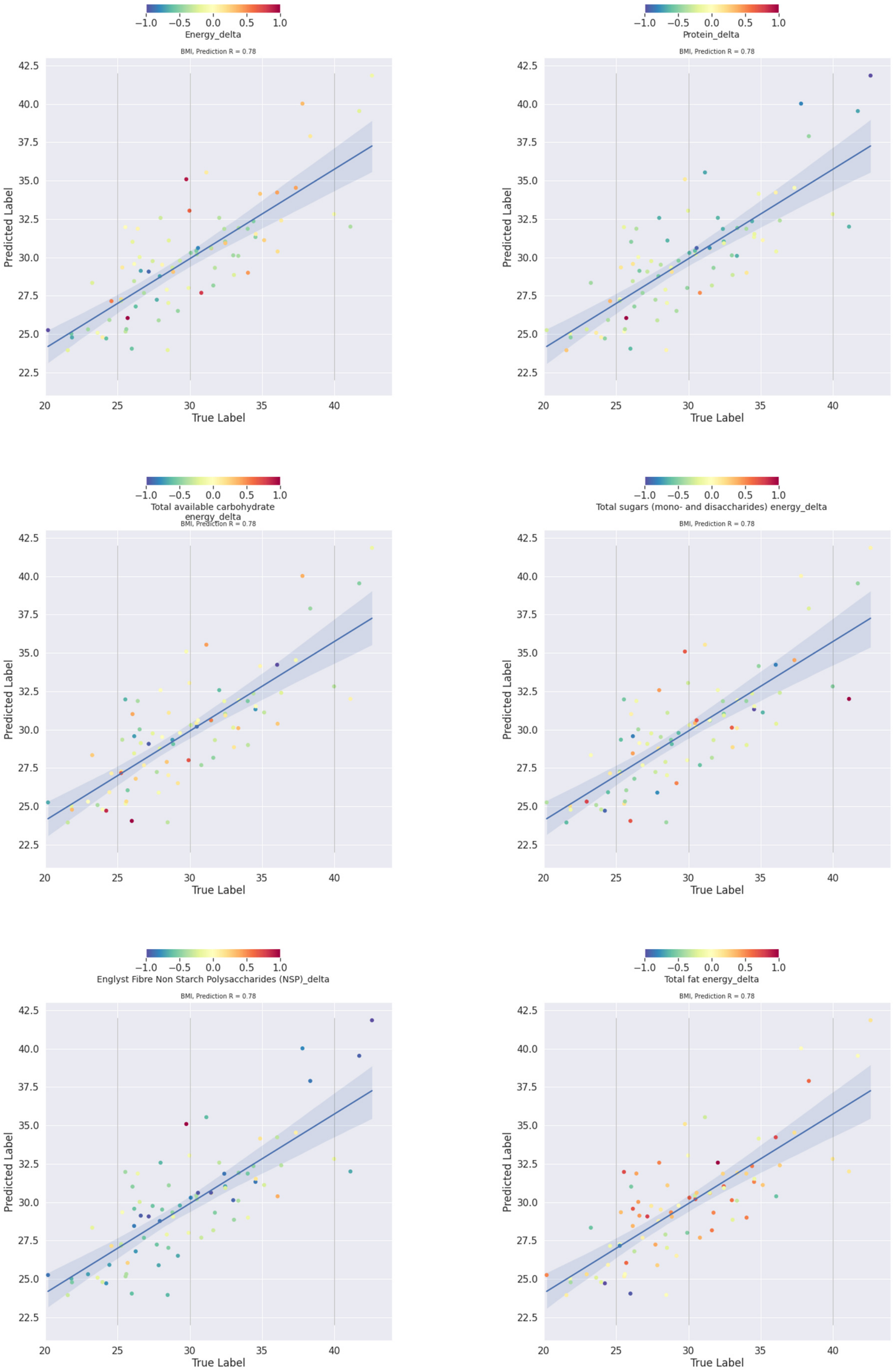

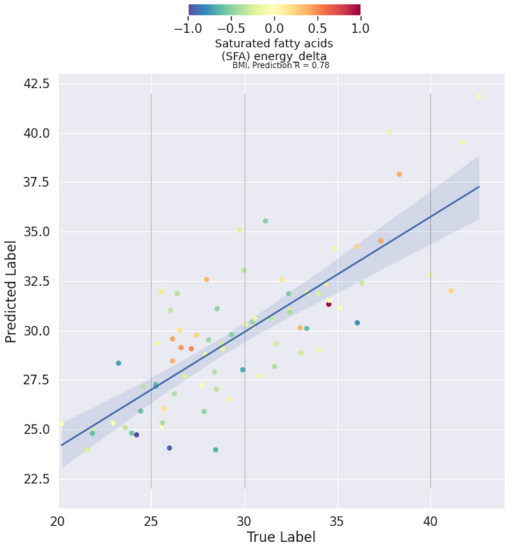

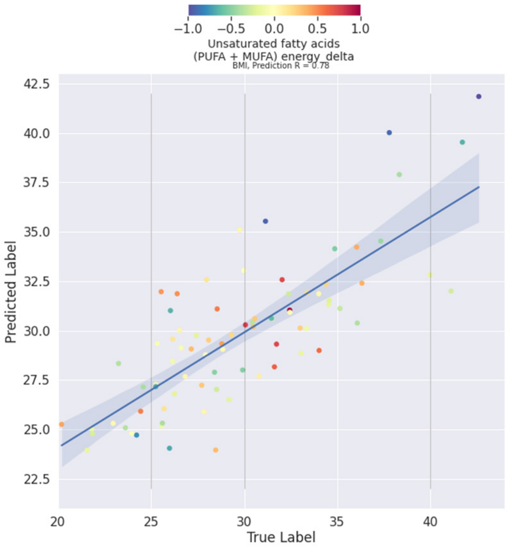

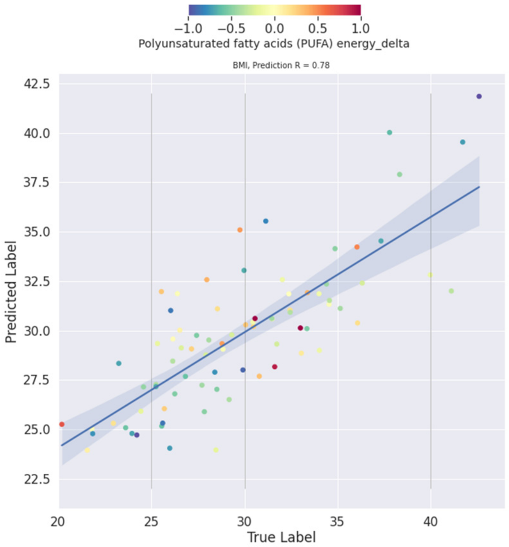

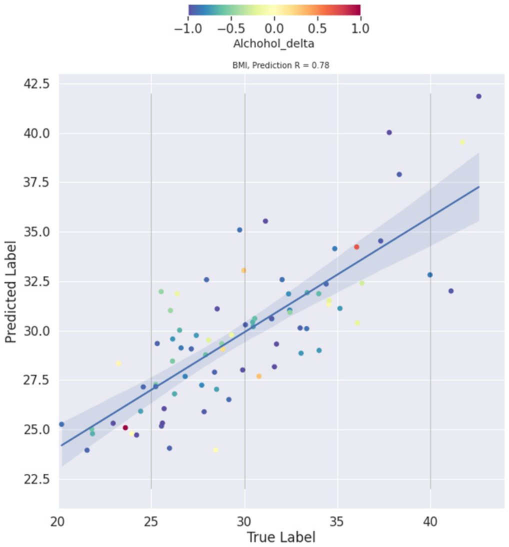

Waist circumference (WC)

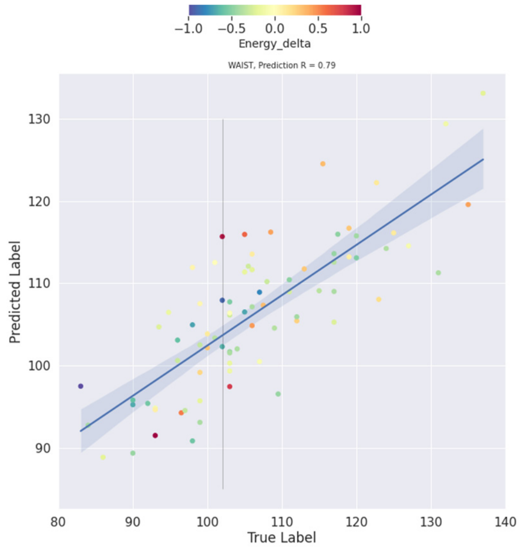

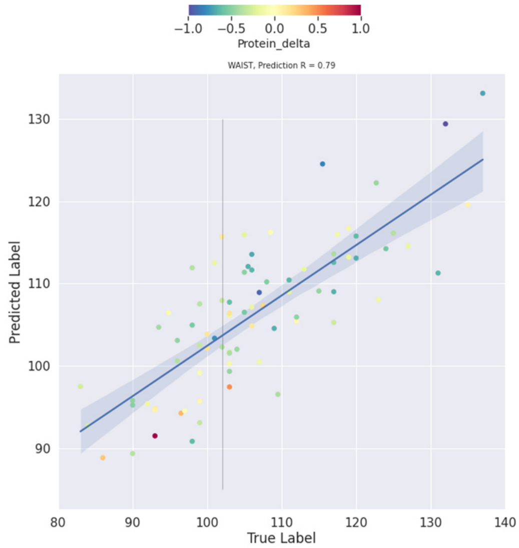

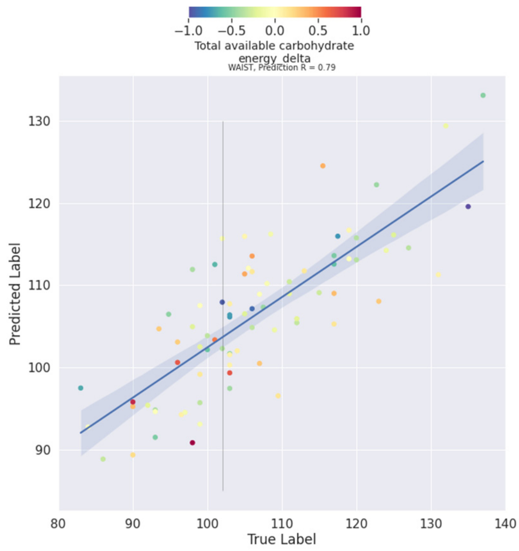
−1.0 −0.5 0.0 0.5 1.0
Total available carbohydrate
energy_delta
WAIST, Prediction R = 0.79
Predicted Label
True Label

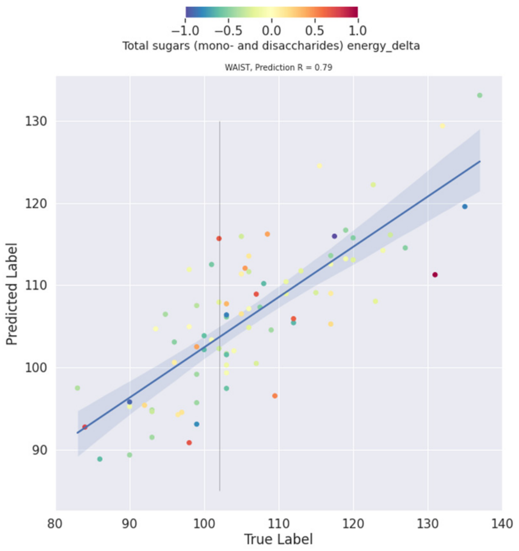
−1.0 −0.5 0.0 0.5 1.0
Total sugars (mono- and disaccharides) energy_delta
WAIST, Prediction R = 0.79
Predicted Label
True Label

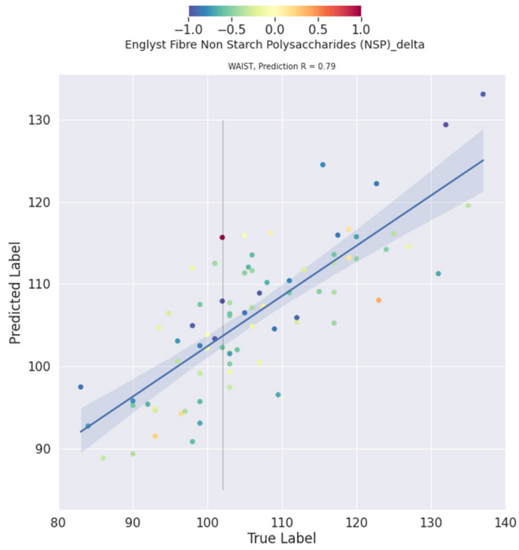
−1.0 −0.5 0.0 0.5 1.0
Englyst Fibre Non Starch Polysaccharides (NSP)_delta
WAIST, Prediction R = 0.79
Predicted Label
True Label

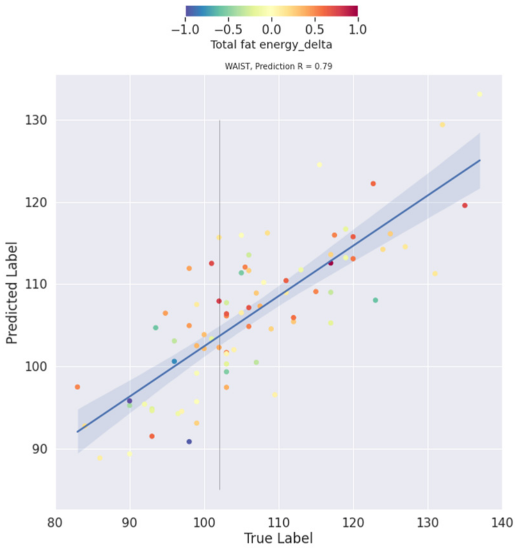
−1.0 −0.5 0.0 0.5 1.0
Total fat energy_delta
WAIST, Prediction R = 0.79
Predicted Label
True Label

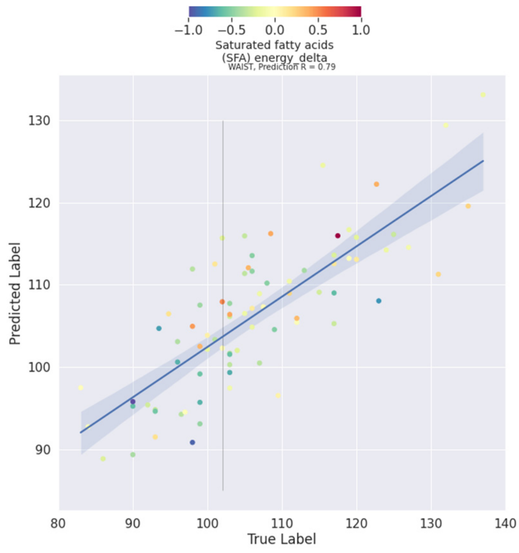
−1.0 −0.5 0.0 0.5 1.0
Saturated fatty acids
(SFA) energy_delta
WAIST, Prediction R = 0.79
Predicted Label
True Label

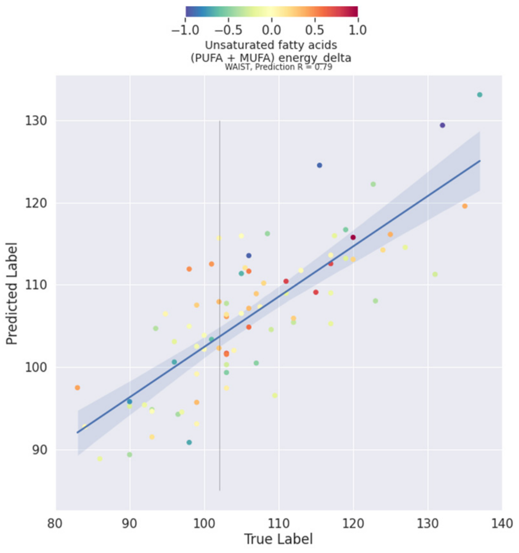
−1.0 −0.5 0.0 0.5 1.0
Unsaturated fatty acids
(PUFA + MUFA) energy_delta
WAIST, Prediction R = 0.79
Predicted Label
True Label

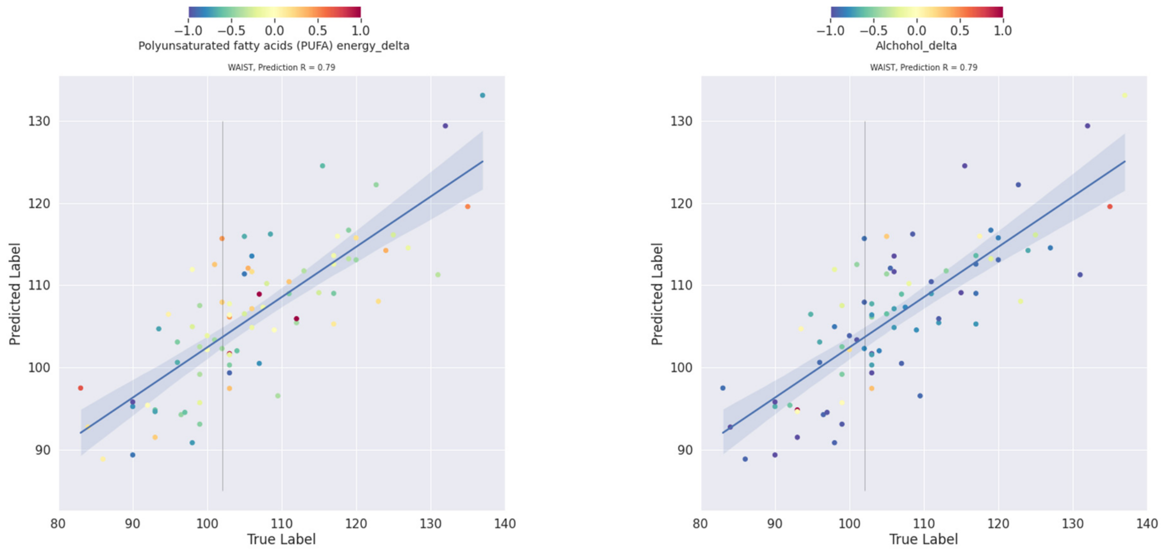

Fasting glucose

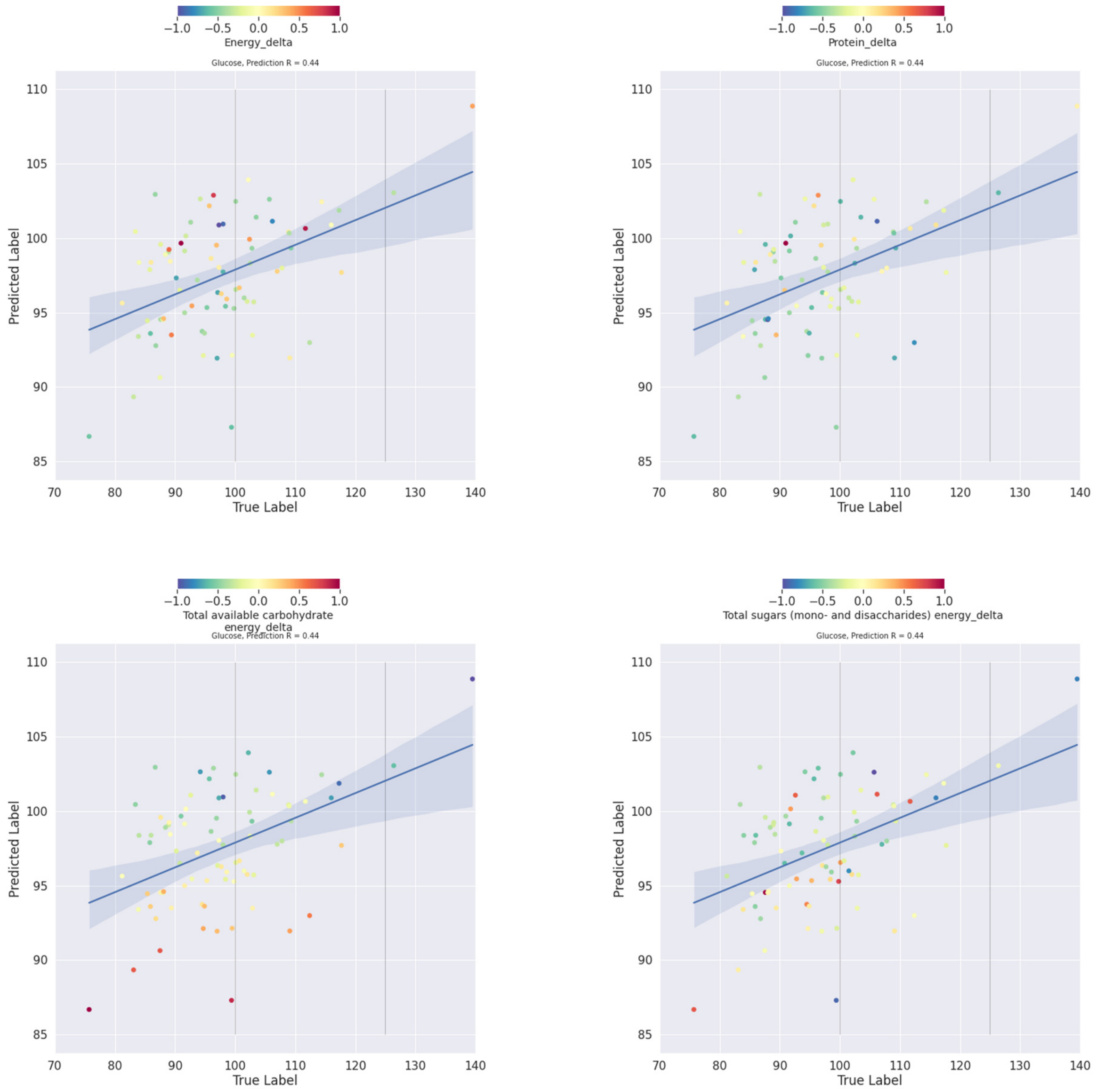

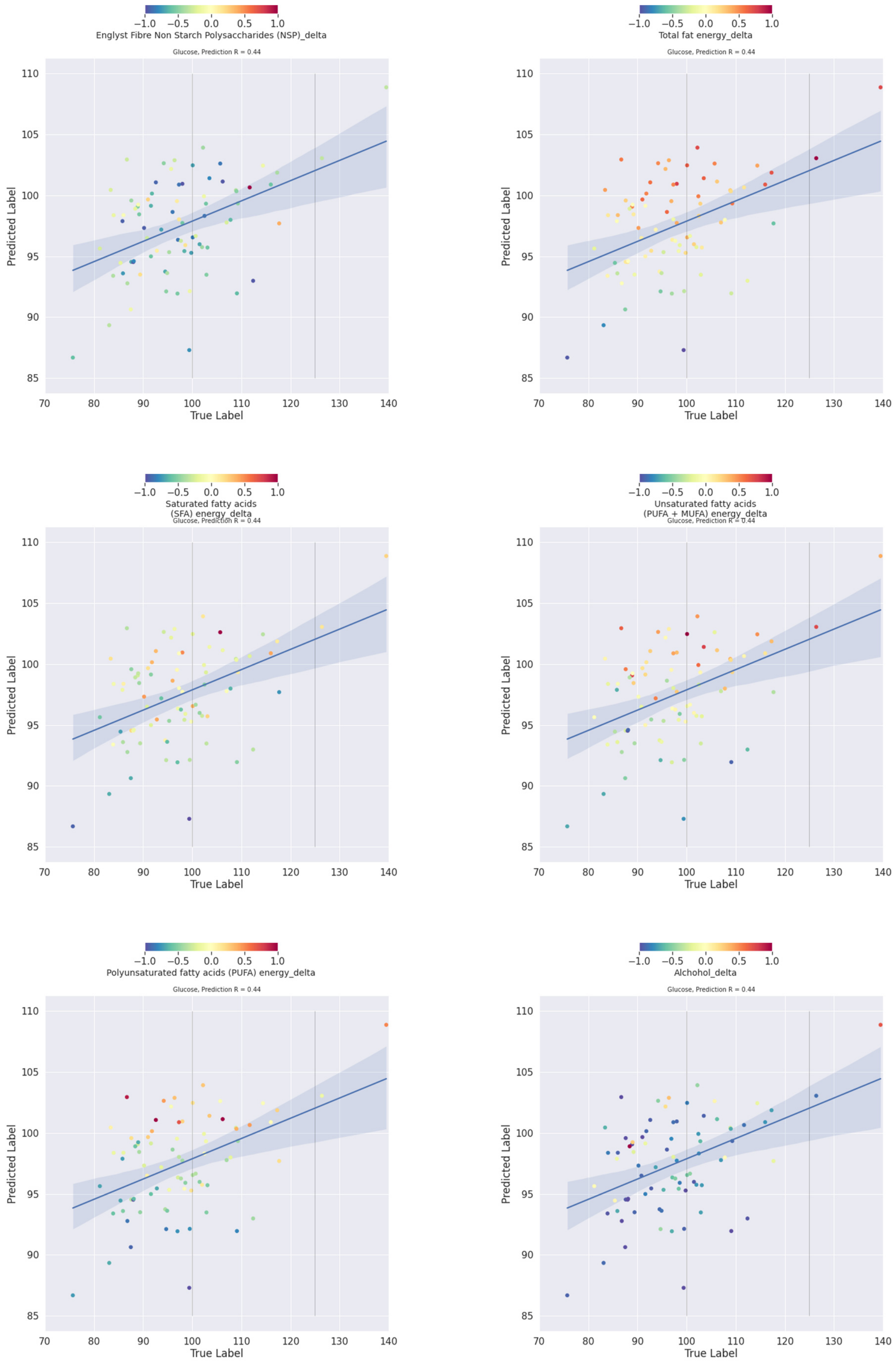
Englyst Fibre Non Starch Polysaccharides (NSP)_delta
Total fat energy_delta
Saturated fatty acids (SFA) energy_delta
Unsaturated fatty acids (PUFA + MUFA) energy_delta
Polyunsaturated fatty acids (PUFA) energy_delta
Alchohol_delta
Glucose, Prediction R = 0.44
Predicted Label
True Label

Fasting insulin

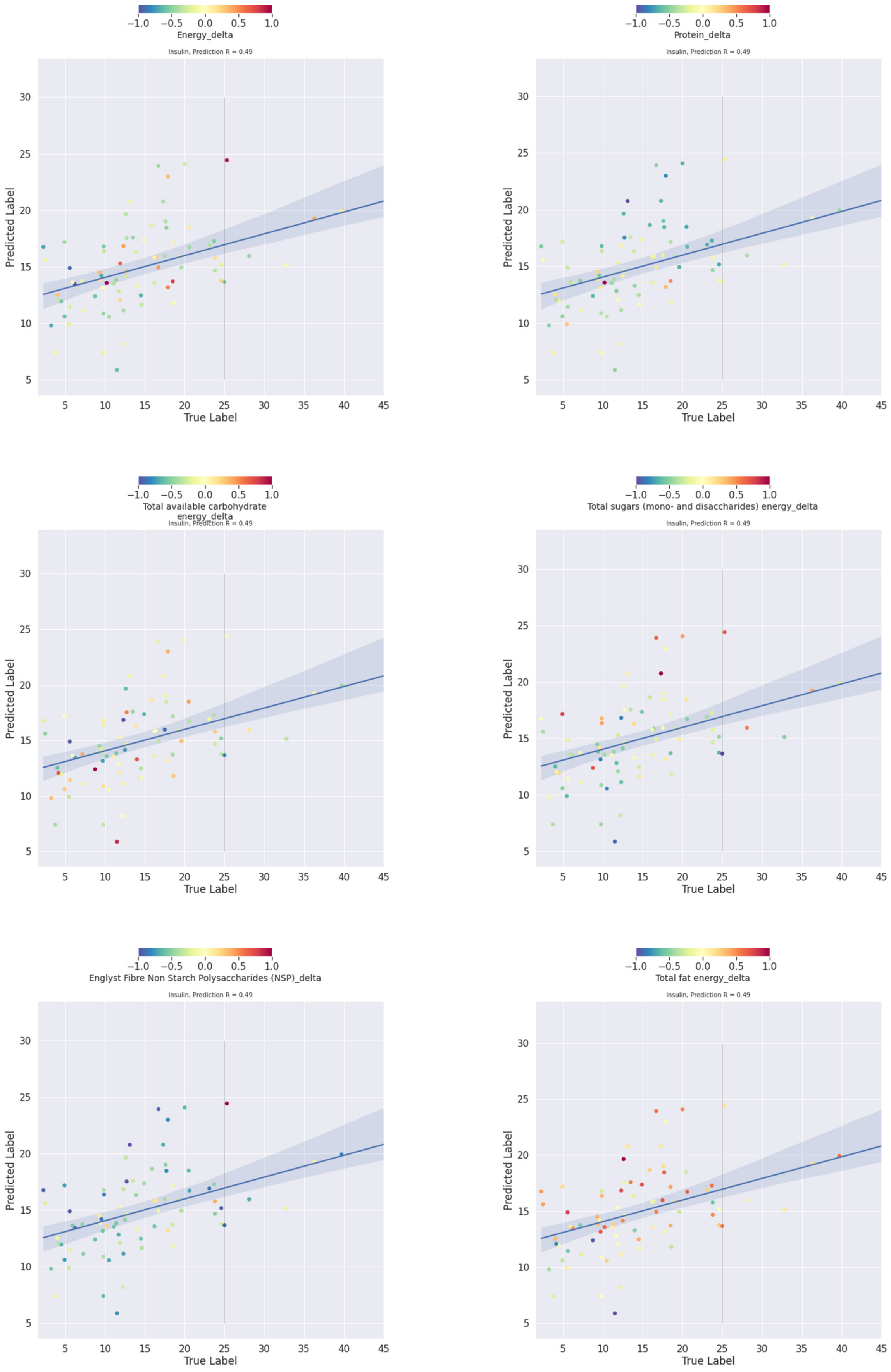

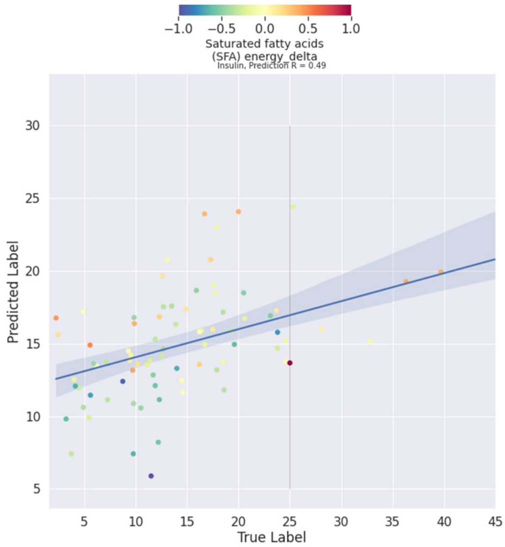

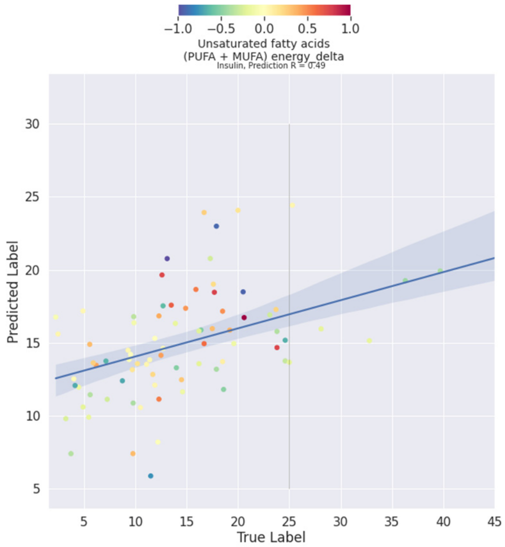

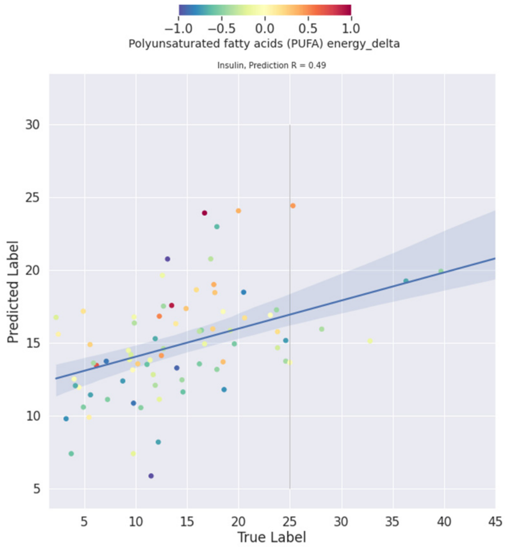

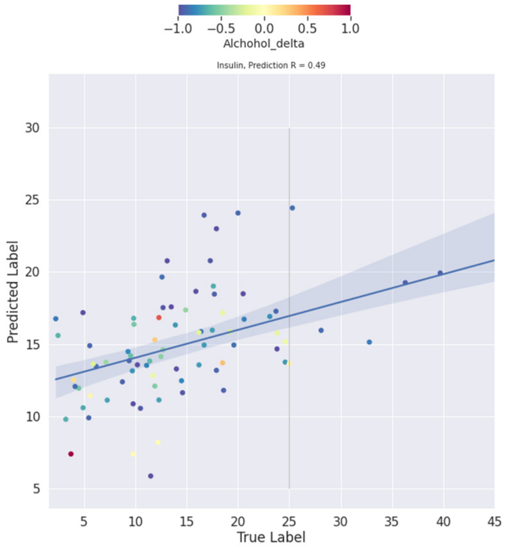

Glycated haemoglobin (HbA1c)

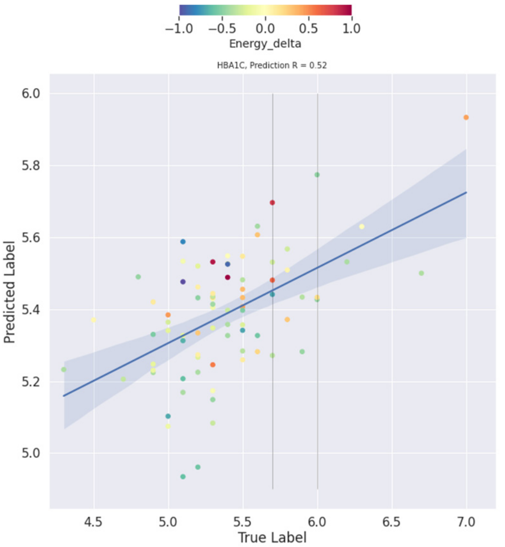

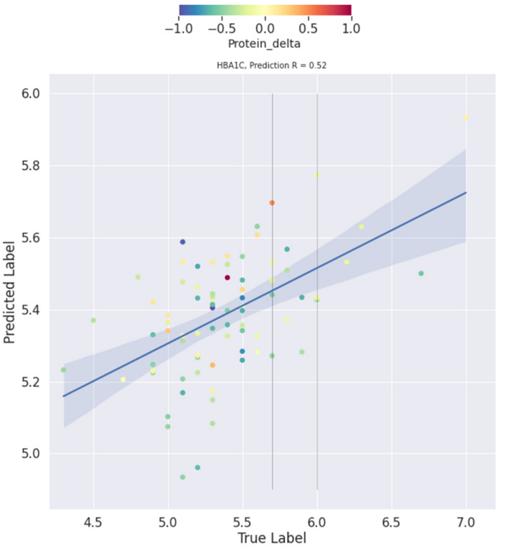

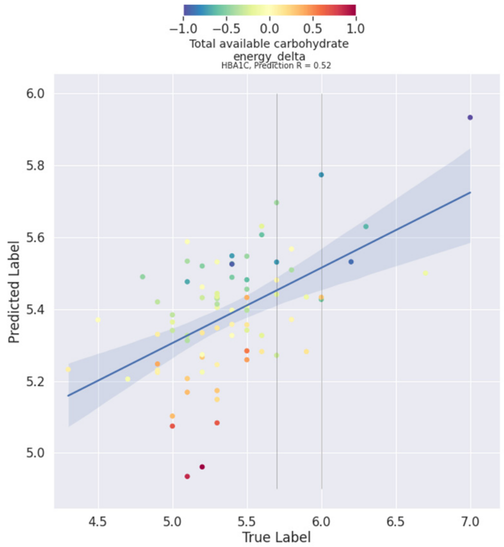
−1.0 −0.5 0.0 0.5 1.0
Total available carbohydrate energy_delta
HBA1C, Prediction R = 0.52
Predicted Label
True Label

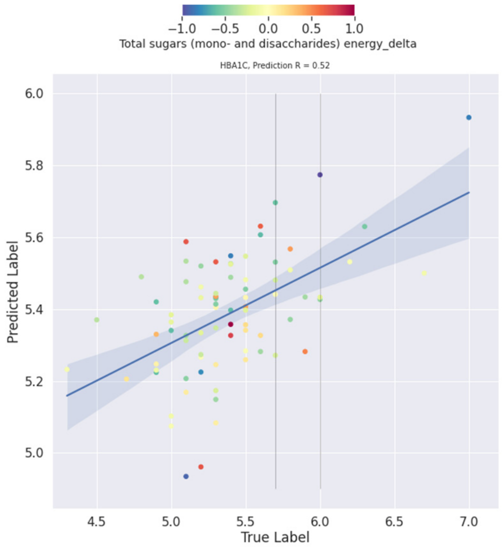
−1.0 −0.5 0.0 0.5 1.0
Total sugars (mono- and disaccharides) energy_delta
HBA1C, Prediction R = 0.52
Predicted Label
True Label

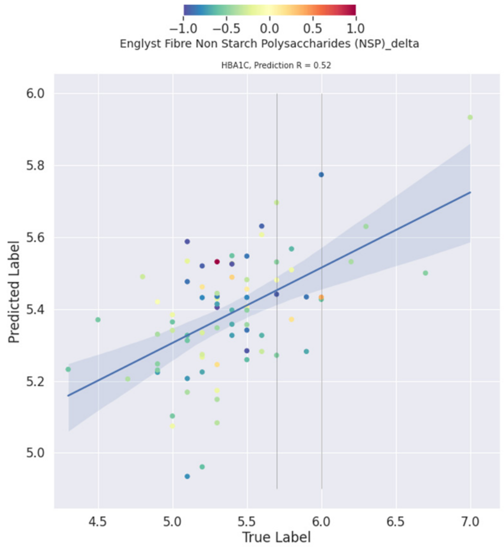
−1.0 −0.5 0.0 0.5 1.0
Englyst Fibre Non Starch Polysaccharides (NSP)_delta
HBA1C, Prediction R = 0.52
Predicted Label
True Label

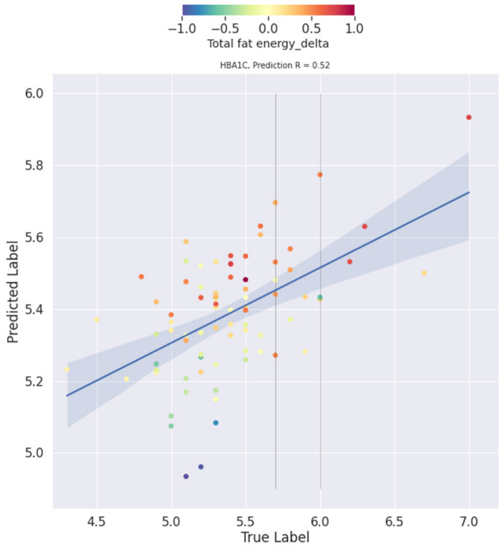
−1.0 −0.5 0.0 0.5 1.0
Total fat energy_delta
HBA1C, Prediction R = 0.52
Predicted Label
True Label

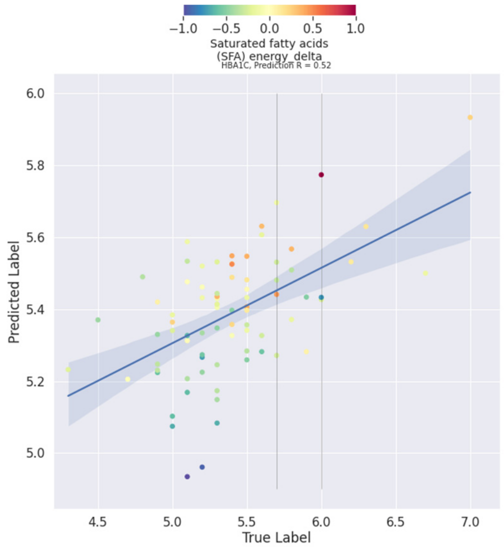
−1.0 −0.5 0.0 0.5 1.0
Saturated fatty acids (SFA) energy_delta
HBA1C, Prediction R = 0.52
Predicted Label
True Label

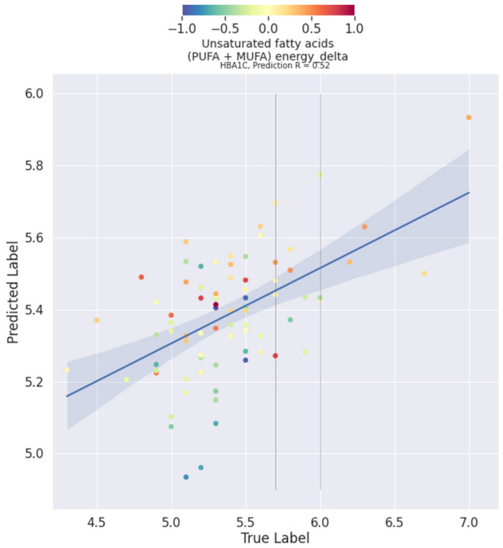
−1.0 −0.5 0.0 0.5 1.0
Unsaturated fatty acids (PUFA + MUFA) energy_delta
HBA1C, Prediction R = 0.52
Predicted Label
True Label

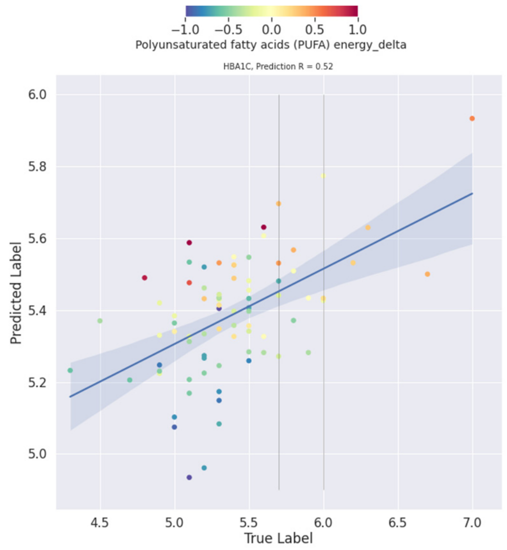

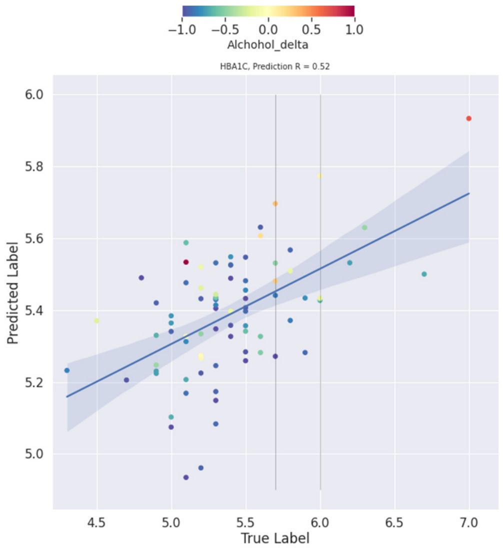

Systolic blood pressure (SBP)

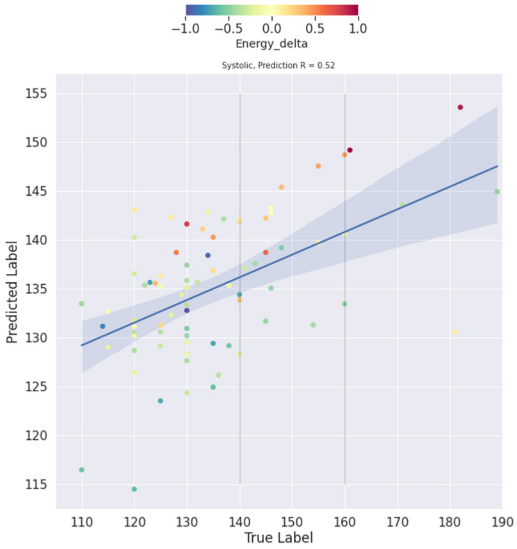

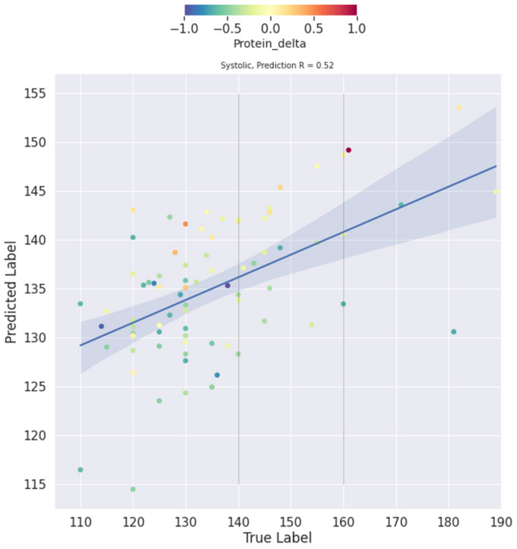

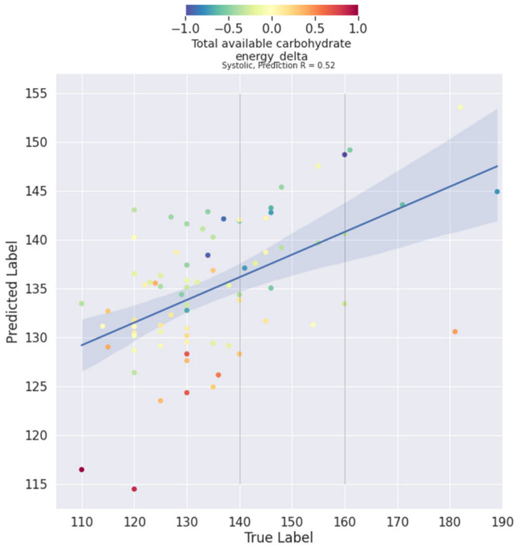

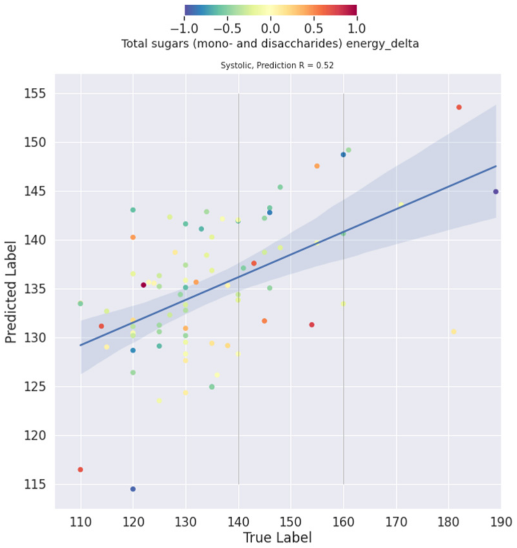

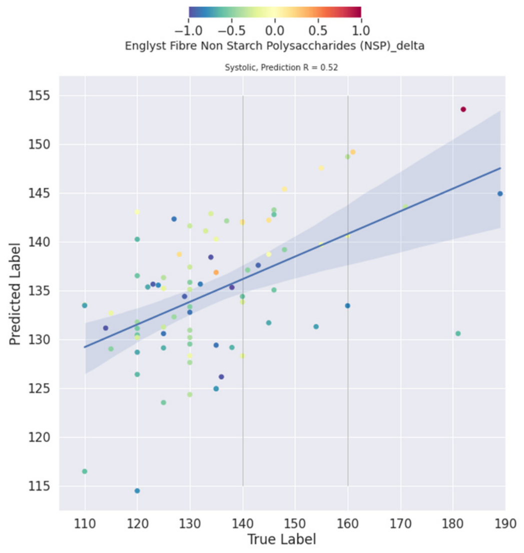
−1.0 −0.5 0.0 0.5 1.0
Englyst Fibre Non Starch Polysaccharides (NSP)_delta
Systolic, Prediction R = 0.52
Predicted Label
True Label

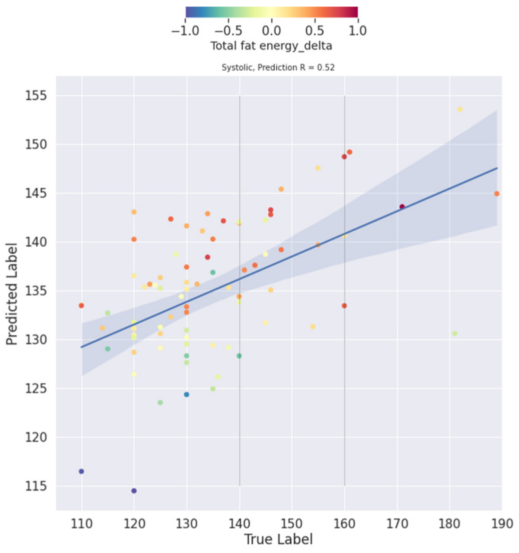
−1.0 −0.5 0.0 0.5 1.0
Total fat energy_delta
Systolic, Prediction R = 0.52
Predicted Label
True Label

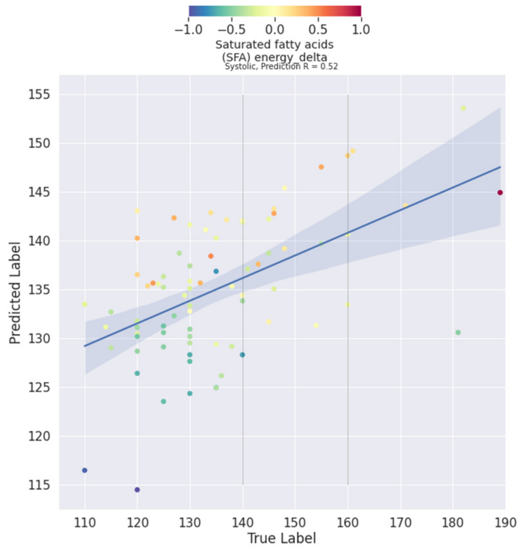
−1.0 −0.5 0.0 0.5 1.0
Saturated fatty acids
(SFA) energy_delta
Systolic, Prediction R = 0.52
Predicted Label
True Label

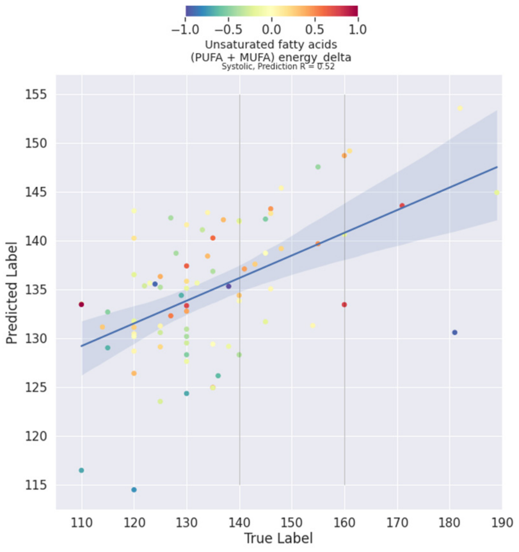
−1.0 −0.5 0.0 0.5 1.0
Unsaturated fatty acids
(PUFA + MUFA) energy_delta
Systolic, Prediction R = 0.52
Predicted Label
True Label

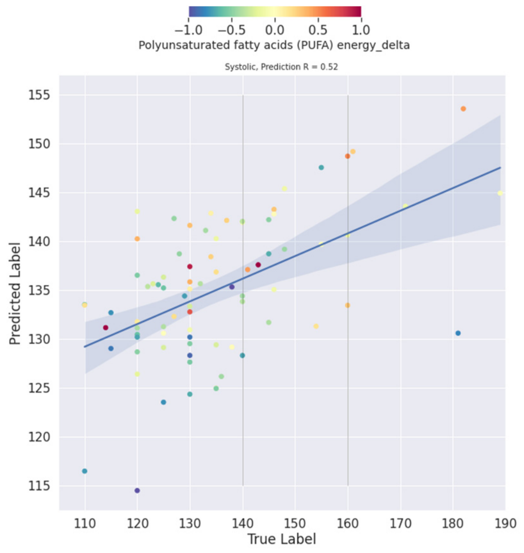
−1.0 −0.5 0.0 0.5 1.0
Polyunsaturated fatty acids (PUFA) energy_delta
Systolic, Prediction R = 0.52
Predicted Label
True Label

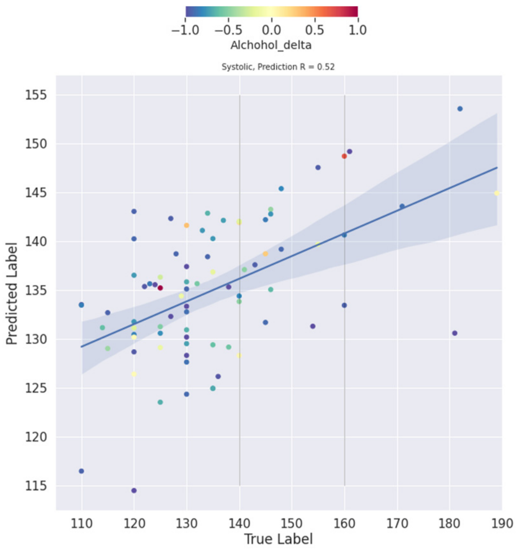
−1.0 −0.5 0.0 0.5 1.0
Alchohol_delta
Systolic, Prediction R = 0.52
Predicted Label
True Label

Figure S2. Magnitude of regression coefficients. High magnitude is related to a significant univariate effect on the prediction of the given target.

Women | Men

Body mass index (BMI)

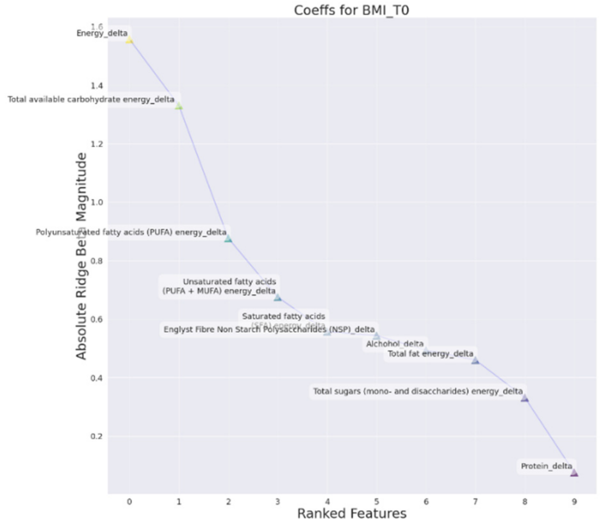

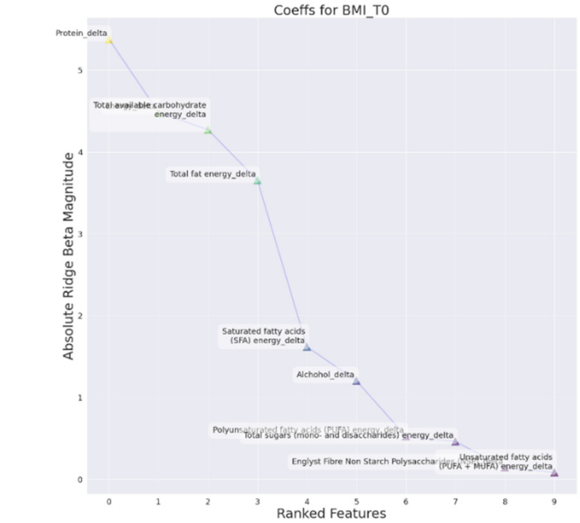

Waist circumference (WC)

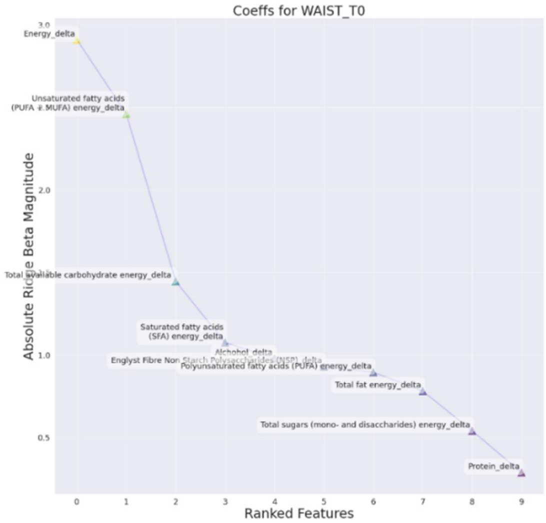

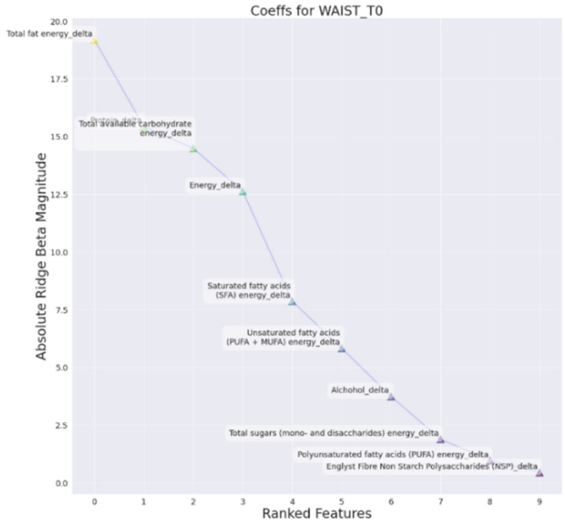

Triglycerides (TG)

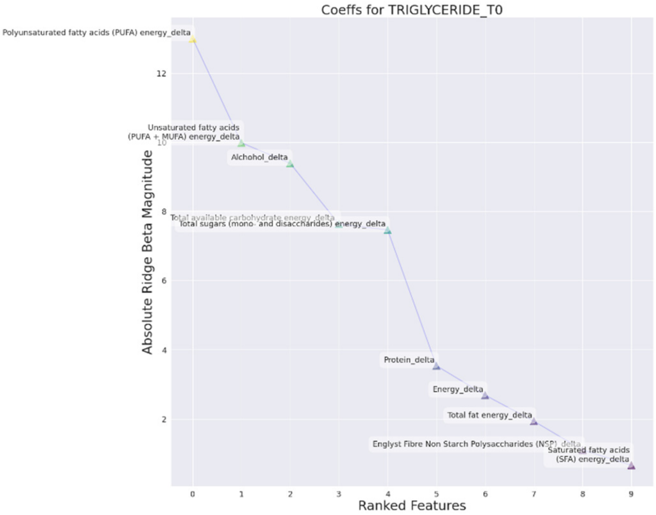

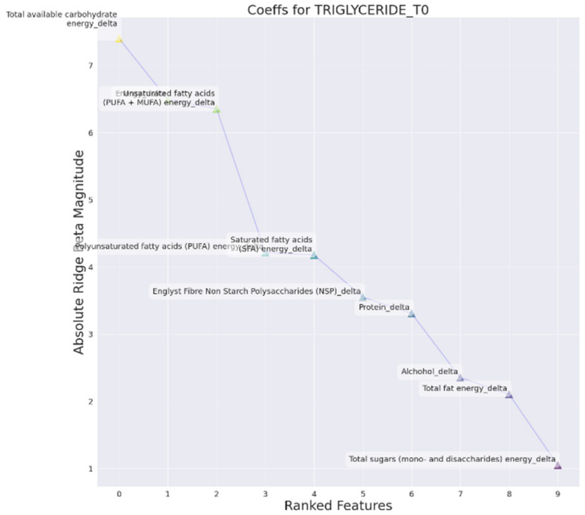

Total cholesterol

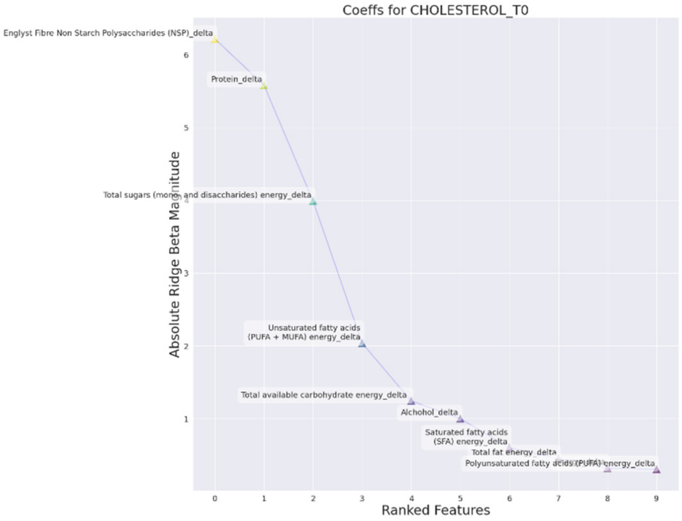

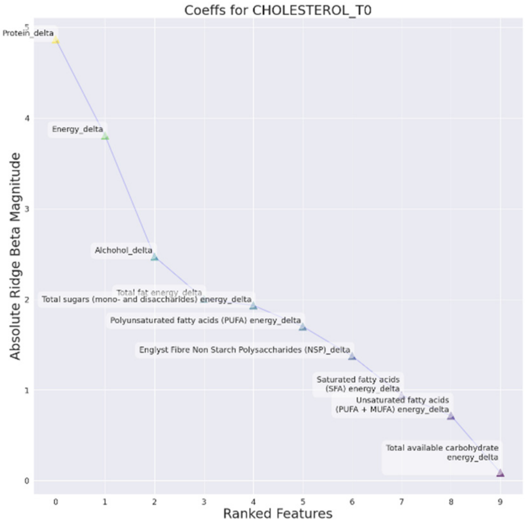

LDL cholesterol (LDL-C)

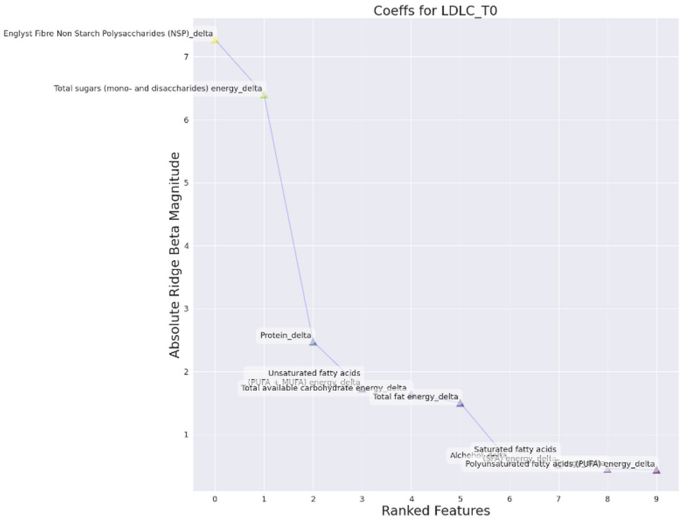

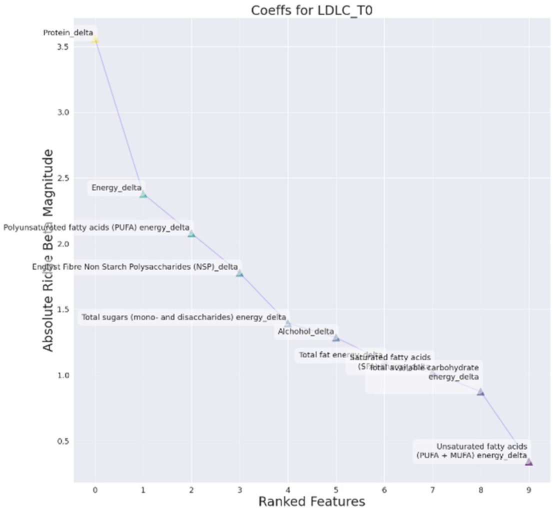

HDL cholesterol (HDL-C)

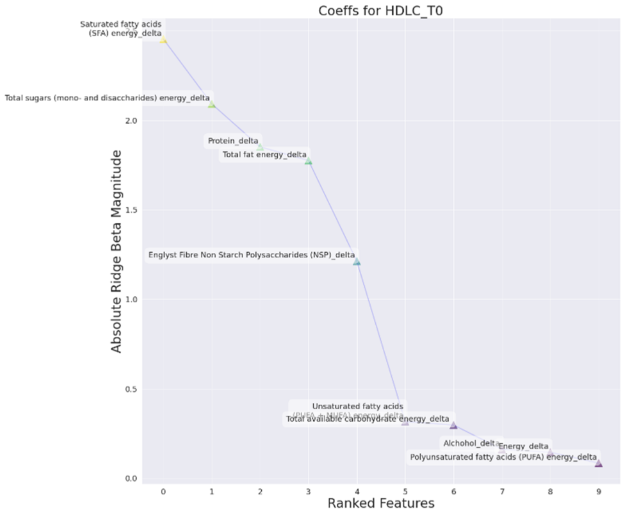

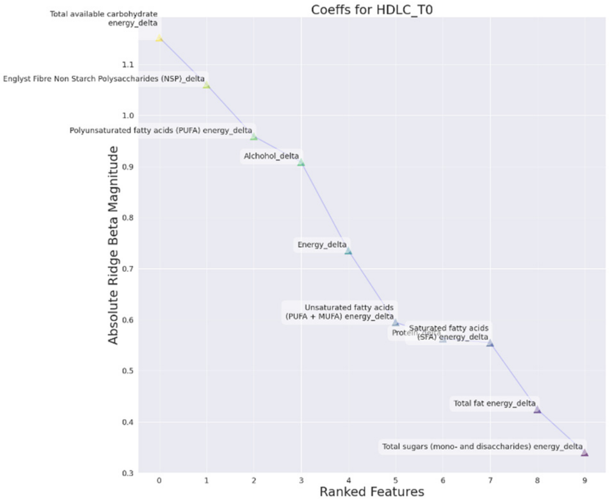

Fasting glucose

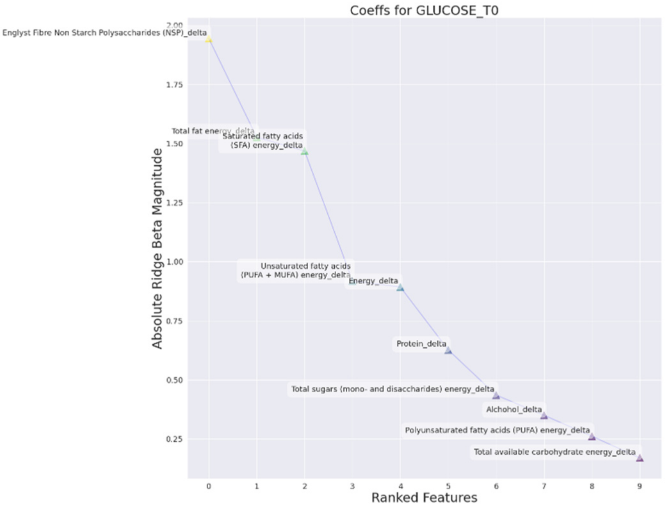

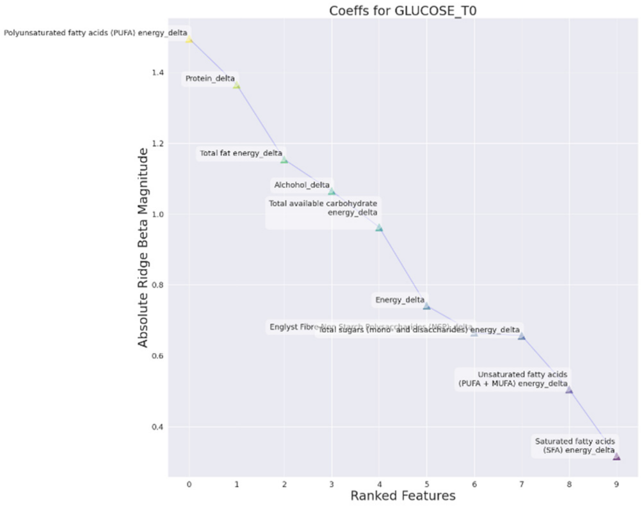

Fasting insulin

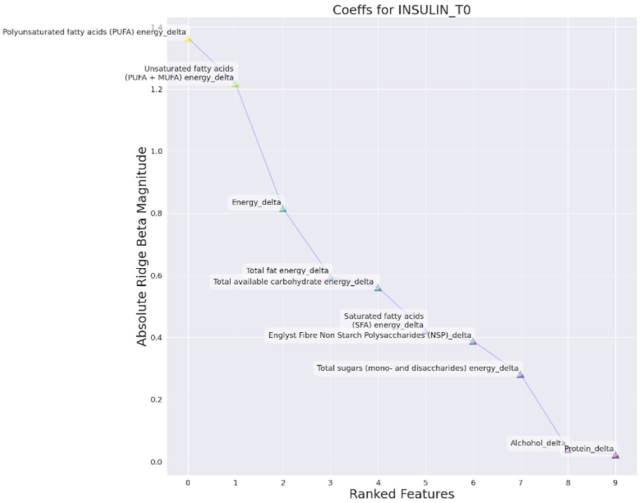

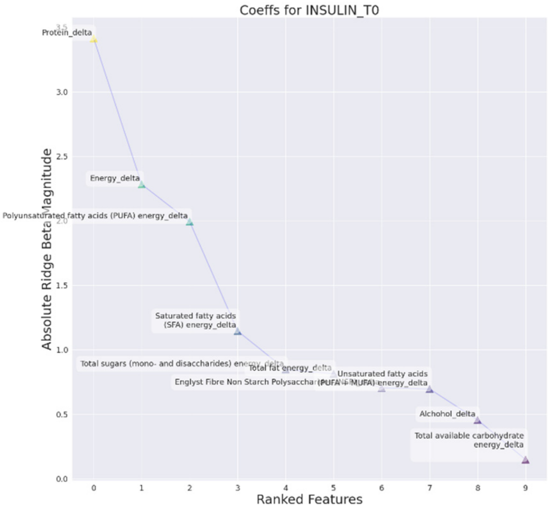

Glycated haemoglobin (HbA1c)

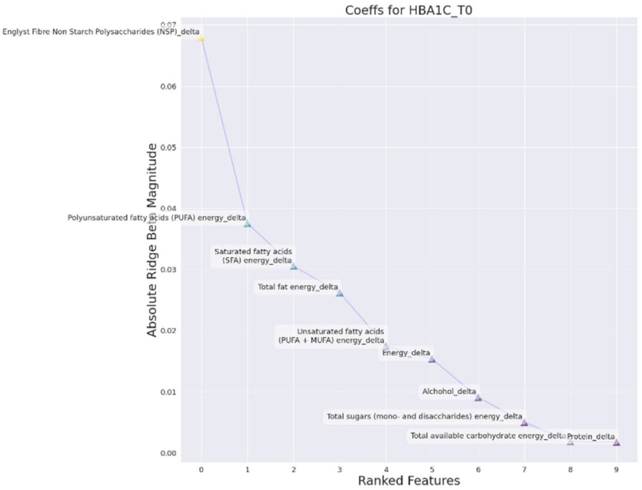

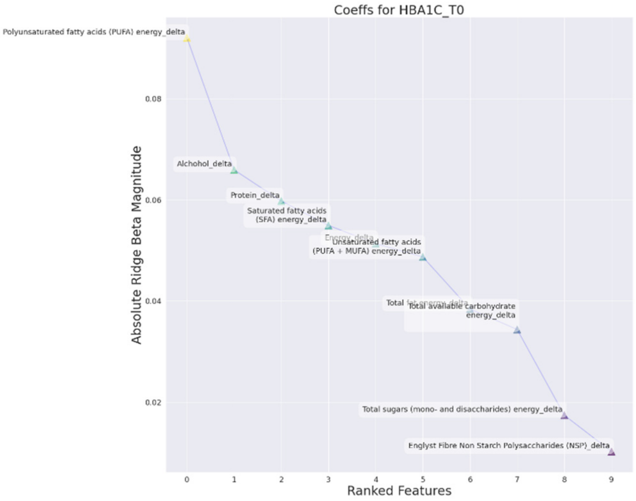

Systolic blood pressure (SBP)

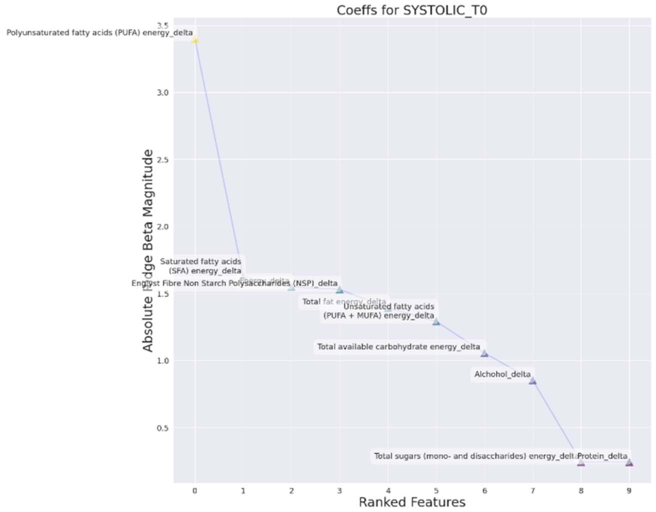

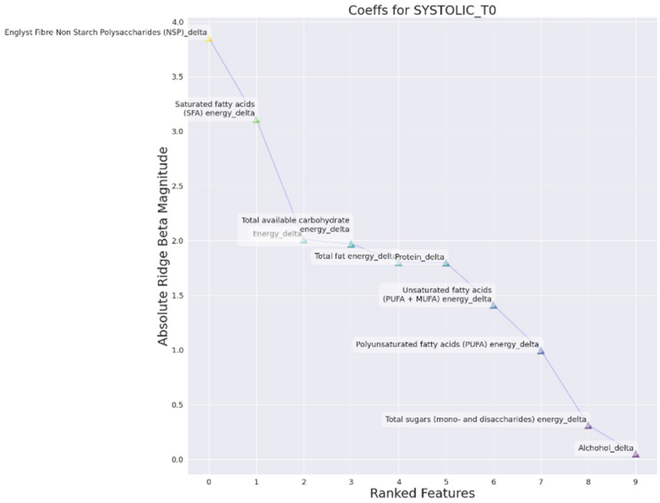

Diastolic blood pressure (DBP)

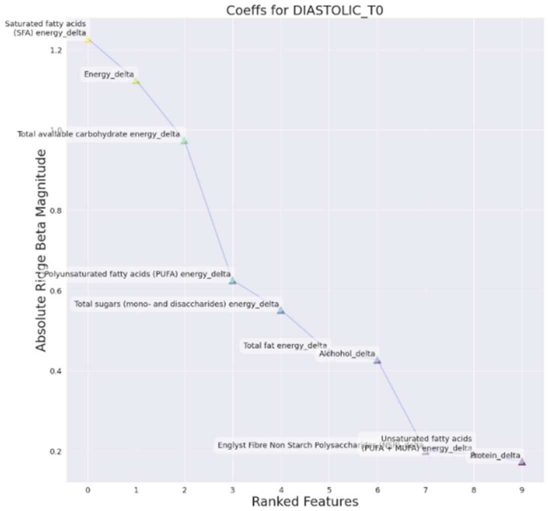

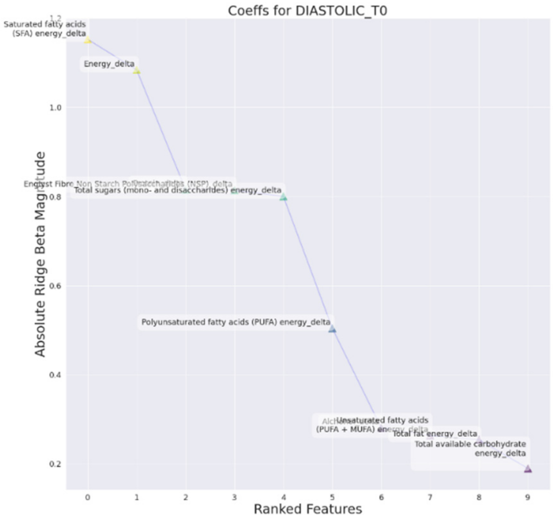

References

1. WHO, *Physical status: The use of and interpretation of anthropometry. Report of a WHO Expert Committee*. World Health Organization: Geneva, Switzerland, 1995.
2. Lipsy, R.J., The National Cholesterol Education Program Adult Treatment Panel III guidelines. *J Manag Care Pharm* **2003**, *9*, 2-5.
3. American Diabetes Association, Classification and diagnosis of diabetes: Standards of medical care in diabetes - 2018. *Diabetes Care* **2018**, *41*, S13-S27.
4. Ambiger, S., Study of insulin resistance and lipid profile in polycystic ovarian syndrome. *Int J Sci Res Publ* **2016**, *6*, 1-5.
5. Mancia, G.; Fagard, R.; Narkiewicz, K.; Redon, J.; Zanchetti, A.; Bohm, M.; Christiaens, T.; Cifkova, R.; De Backer, G.; Dominiczak, A., *et al.*, 2013 ESH/ESC Guidelines for the management of arterial hypertension: The Task Force for the management of arterial hypertension of the European Society of Hypertension (ESH) and of the European Society of Cardiology (ESC). *J Hypertens* **2013**, *31*, 1281-1357.
